# Structural basis for dynamic mechanism of nitrate/nitrite antiport by NarK

**DOI:** 10.1038/ncomms8097

**Published:** 2015-05-11

**Authors:** Masahiro Fukuda, Hironori Takeda, Hideaki E. Kato, Shintaro Doki, Koichi Ito, Andrés D. Maturana, Ryuichiro Ishitani, Osamu Nureki

**Affiliations:** 1 Department of Biological Sciences, Graduate School of Science, The University of Tokyo, 7-3-1 Hongo, Bunkyo-ku, Tokyo 113-0033, Japan; 2 Global Research Cluster, RIKEN, 2-1 Hirosawa, Wako-shi, Saitama 351-0198, Japan; 3 Department of Medical Genome Sciences, Graduate School of Frontier Sciences, The University of Tokyo, 5-1-5 Kashiwanoha, Kashiwa-shi, Chiba 277-8562, Japan; 4 Department of Bioengineering Sciences, Graduate School of Bioagricultural Sciences, Nagoya University, Furo-cho, Chikusa-ku, Nagoya 464-8601, Japan

## Abstract

NarK belongs to the nitrate/nitrite porter (NNP) family in the major facilitator superfamily (MFS) and plays a central role in nitrate uptake across the membrane in diverse organisms, including archaea, bacteria, fungi and plants. Although previous studies provided insight into the overall structure and the substrate recognition of NarK, its molecular mechanism, including the driving force for nitrate transport, remained elusive. Here we demonstrate that NarK is a nitrate/nitrite antiporter, using an *in vitro* reconstituted system. Furthermore, we present the high-resolution crystal structures of NarK from *Escherichia coli* in the nitrate-bound occluded, nitrate-bound inward-open and *apo* inward-open states. The integrated structural, functional and computational analyses reveal the nitrate/nitrite antiport mechanism of NarK, in which substrate recognition is coupled to the transport cycle by the concomitant movement of the transmembrane helices and the key tyrosine and arginine residues in the substrate-binding site.

## Abstract (web summary)

Nitrate/nitrite porters (NNP) play a central role in nitrate uptake in archaea, bacteria, fungi and plants. Here, Fukuda *et al*. use a liposome-based transport assay, X-ray crystallography and molecular dynamics simulation to reveal the dynamic nitrate/nitrite antiport mechanism of a bacterial NNP, NarK.

## Graphical abstract

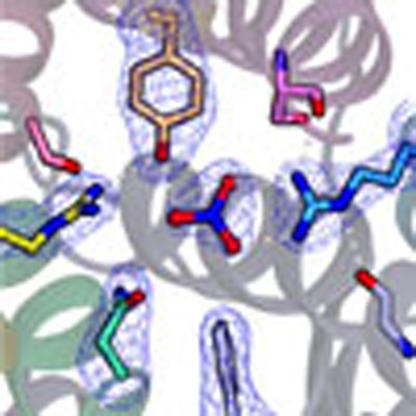

Nitrogen is an essential element for all organisms and is present in numerous biological molecules, including DNA, RNA and protein. Although nitrogen is very abundant in the atmosphere, it is mostly present in the gaseous form, and therefore is not available to most organisms, except for diazotrophs^1^,^2^. Most organisms utilize nitrate, nitrite and ammonium ions as inorganic nitrogen sources. Nitrate respiration and assimilation are the principal processes in the nitrogen cycle of the ecosystem. In bacteria, nitrate is used as a substrate for anaerobic nitrate respiration^3^. Plants growing in aerobic soil absorb nitrate from the roots and assimilate it as a nutrient^4^. The first step of nitrate respiration and assimilation is cellular nitrate uptake, which is catalysed by nitrate transporter families; that is, the ABC transporter superfamily^5^, the Nitrate transporter 1/Peptide transporter Family (NPF)^6^,^7^ and the nitrate/nitrite porter (NNP) family. Although both the NNP and NPF families belong to the major facilitator superfamily (MFS), they share low amino-acid sequence similarity. The NNP family is the most prevalent nitrate transporter group conserved in archaea, bacteria, fungi, yeast, algae and plants^8^,^9^. The NNP family members in higher plants, such as NRT2.1 from *Arabidopsis thaliana*, are predominantly expressed in root cells, where they play important roles in nitrogen assimilation as key components of a high-affinity transport system for nitrate^10^. The bacterial NNP family transporters play an important role in the uptake of nitrate as a terminal electron acceptor for anaerobic respiration, and the extrusion of cytotoxic nitrite produced by anaerobic respiration^11^. In *Escherichia coli*, there are two NNP family transporters, NarK and NarU. NarK is highly expressed during anaerobic growth in the presence of nitrate, and plays a central role in nitrate uptake, while NarU has important functions during severe nutrient starvation or slow growth^12^. NarK and NarU share 75% amino-acid sequence similarity, suggesting that they have a similar transport mechanism.

Despite the importance of the NNP family transporters, their molecular-level functions are still under debate. Several hypotheses for the functions of NarK and NarU have been proposed: nitrate/nitrite antiporter^13^,^14^, nitrite/H^+^ antiporter^11^,^15^,^16^, nitrate/H^+^ symporter^15^,^16^,^17^, simple nitrite uniporter^15^,^17^ and Na^+^- and/or K^+^-coupled nitrate and/or nitrite transporter^18^. The lack of facile nitrogen and oxygen radioisotopes has hampered the direct measurement of their transport activity using *in vitro* reconstituted systems, and thus has limited an understanding of the precise functions of the NNP family transporters.

Recently, the crystal structures of NarK and NarU from *E. coli* were reported, at resolutions of 2.6–2.8 and 3.0–3.1 Å, respectively^18^,^19^. These structures revealed that both NarK and NarU contain 12 transmembrane (TM) helices with pseudo twofold symmetry axes, similar to other MFS transporters, and suggested that they transport substrates via an alternating-access mechanism^20^, in which the transporter adopts several conformations, involving outward-open, occluded and inward-open states, during the transport cycle. Furthermore, these structures provided insights into the substrate recognition^18^,^19^. However, the molecular mechanism of the NNP family transporters remains largely unknown because of our poor understanding of their molecular-level functions and the lack of the atomic-resolution structures. Recently, the structures of another nitrate transporter, NRT1.1 from *A. thaliana*, in the NPF family, were reported^21^,^22^; however, its substrate recognition mechanism is entirely different from that of the NNP family, and thus the molecular mechanism of the NNP family remains elusive.

In this work, we demonstrate that NarK is a nitrate/nitrite antiporter, using an *in vitro* reconstituted system. Furthermore, to elucidate the precise molecular mechanism of nitrate/nitrite transport by NarK, we determined the crystal structures of NarK from *E. coli* in three different states (that is, nitrate-bound occluded, nitrate-bound inward-open and *apo* inward-open states) at 2.35–2.4 Å resolutions. In combination with *in vivo* and *in vitro* functional analyses and molecular dynamics (MD) simulations, our results show the molecular mechanism of nitrate/nitrite antiport by NarK, in which the key tyrosine and arginine residues play essential roles in the coupling mechanism between the structural changes and nitrate/nitrite recognition.

## Results

### *In vitro* functional analysis of NarK

**Figure 1:**
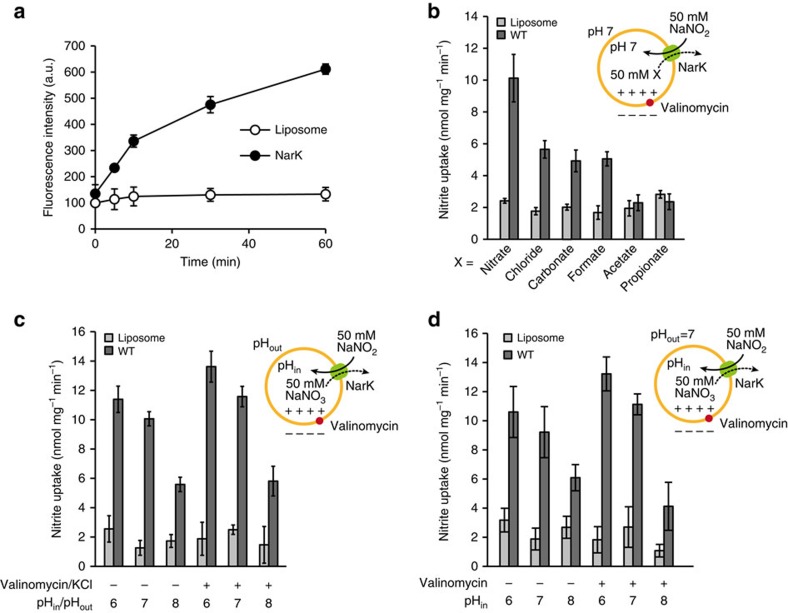
Liposome-based nitrite-flux assay of NarK. (**a**) The time-dependent nitrite influx driven by the nitrate concentration gradient across the membrane at pH 7, in the presence of a membrane potential, measured at 37 °C. The intra- and extraliposomal solutions contained 50 mM NaNO_3_ and NaNO_2_, respectively. (**b**) Substrate specificity of NarK. The intraliposomal solution contained 50 mM NaNO_3_, NaCl, Na_2_CO_3_, HCOONa, CH_3_COONa or C_2_H_5_COONa. The extraliposomal solution contained 50 mM NaNO_2_ and was the same in all measurements. (**c**) pH and membrane-potential dependence of NarK activity. (**d**) Liposome-based nitrite-uptake assay in the presence of pH concentration gradients across the membrane. All error bars represent the s.d. of three independent trials.

To understand the exact function of NarK, we developed an *in vitro* assay system using NarK-reconstituted proteoliposomes and the fluorescent probe, 4-amino-5-methylamino-2′,7′-difluorescein (DAF-FM)^23^. Although DAF-FM was originally developed as a fluorescent probe for nitric oxide, it also reacts with nitrite under acidic conditions to form a fluorescent compound, triazolofluorescein, but not with other ions, including nitrate^23^ (Supplementary Fig. 1a,b). In this system, we quantified the amount of nitrite accumulated in the liposomes by measuring the fluorescence derived from the formation of triazolofluorescein. We observed the uptake of nitrite when nitrate was present inside the liposomes (Fig. 1a). In contrast, almost no nitrite uptake was observed when either acetate or propionate, which are larger than nitrate, was inside the liposomes (Fig. 1b). Anions with similar or smaller sizes than nitrate; that is, chloride, carbonate and formate, exhibited weaker nitrite-uptake activities than nitrate (Fig. 1b). While the lower pH resulted in increased transport activity in spite of the presence or absence of the membrane potential (Fig. 1c), the pH gradient across the membrane had no effect on the activity (Fig. 1d), suggesting that the H^+^ concentration gradient across the membrane does not drive the substrate transport. Taken together, the flux of nitrite occurs in the presence of nitrate (and its near-cognate anions) as a counteranion, which is transported towards the outside of the liposomes. Although our results do not preclude the possibility that NarK is driven by H^+^, they clearly demonstrate that NarK is a nitrate/nitrite antiporter.

### Overall structure

**Figure 2:**
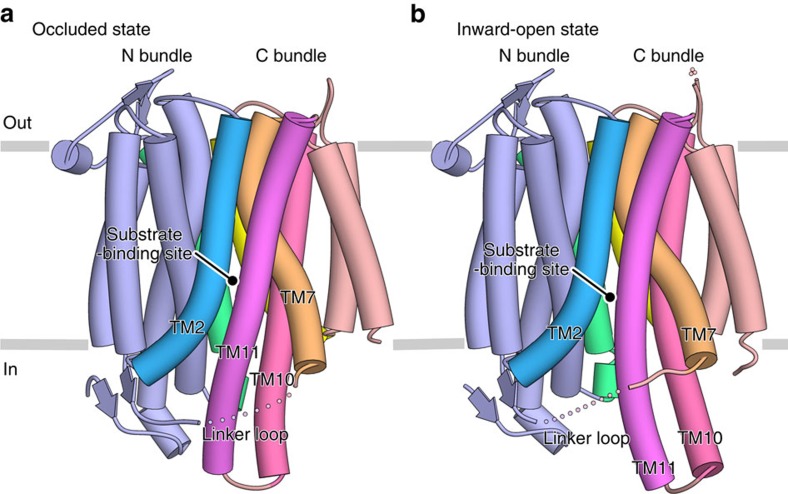
Overall structure of *E. coli* NarK. (**a**,**b**) Overall structures of NarK in the occluded (**a**) and inward-open (**b**) states. Each molecule contains 12 TM helices, forming the N bundle (TM1–6) and the C bundle (TM7–12). TM2, TM5, TM7, TM8, TM10 and TM11 are coloured light blue, green, amber, yellow, pink and purple, respectively. Other TM helices in the N and C bundles are coloured pale blue and light pink, respectively. Grey bars indicate the approximate location of the lipid bilayer. The figures depicting the molecular structures were prepared using CueMol (http://www.cuemol.org/).

To elucidate the structural basis for the nitrate/nitrite antiport mechanism of NarK, we determined the crystal structures of NarK from *E. coli*. Three distinct types of crystals (I, II and III) were obtained, using the lipidic cubic phase (LCP) method^24^. The phases were determined with the single isomorphous replacement with anomalous scattering (SIRAS) method, using the mercury-derivative crystal. We finally determined the crystal structures of NarK in three different states—the nitrate-bound occluded, nitrate-bound inward-open and *apo* inward-open states—at resolutions of 2.4, 2.4 and 2.35 Å, respectively (Table 1). Each asymmetric unit of Crystals I and II contained one inward-open molecule, while in the asymmetric unit of Crystal III, there were two occluded molecules (Mol A and B). Since Mol A and B have almost the same structure (r.m.s.d. of 0.38 Å for all Cα atoms), we use Mol A in the following discussion. The overall structures of NarK in both the occluded and inward-open states adopt a canonical MFS-fold with 12 TM helices, with TM1–TM6 and TM7–TM12 forming the N and C bundles, respectively (Fig. 2a,b). These N and C bundles are connected with a linker loop between TM6 and TM7, which is disordered in the crystal structures (Fig. 2a,b). These overall structural features are consistent with the previously reported structures of NarK^19^ and NarU^18^. The substrate-binding site is located at the interface between the N and C bundles, in the middle of the TM segment (Fig. 2a,b). While the substrate-binding site is accessible from the cytoplasmic side in the inward-open structures, it is completely sequestered in the occluded state structure (Supplementary Fig. 2a–c).

### Structural change of the TM helices in the C bundle

**Figure 3:**
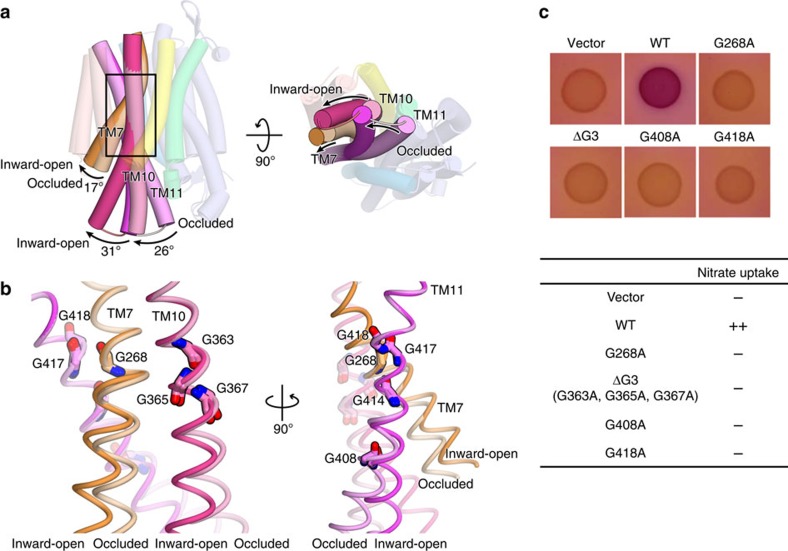
Structural comparison of the occluded and inward-open states. (**a**) Overall structures of NarK in the occluded and inward-open states, viewed from the plane of the membrane (left panel) and the cytoplasmic side (right panel). The two structures were superimposed based on the Cα atoms of the N bundle residues (1–233). The colours of the helices in the inward-open state correspond to those in Fig. 2a,b. TM7, TM10 and TM11 in the occluded state are depicted with pale colours. The rectangle indicates the region highlighted in **b**. (**b**) Close-up views around the conserved Gly residues in TM7, TM10 and TM11. The occluded and inward-open structures were superimposed as in **a**. The Gly residues are shown in stick models. (**c**) Genetic analysis of the nitrate transport activity of NarK mutants of the conserved Gly residues involved in the bending of TM7, TM10 and TM11, using the no-induction system. *E. coli* cultures were spotted on MacConley-glucose-TMAO agar plates under anaerobic conditions, and their colours were monitored. *E. coli* cells expressing intact NarK accumulate nitrate, generating dark-red spots, while those expressing inactive NarK generate pale-yellow spots (see Methods). The results of this genetic analysis are summarized in the table on the right.

While no structural difference between the occluded and inward-open structures was observed in the N bundle, we observed a large structural change of the TM helices in the C bundle. The TM10 and TM11 helices are straight in the occluded structure, whereas they are bent around the conserved Gly residues (G363, G365, G367, G408, G414, G417 and G418) in the inward-open structures (Fig. 3a,b and Supplementary Fig. 3). Among them, G408, G414, G417 and G418 are included in the conserved Nitrate Signature motif 2 (refs 25, 26). The cytosolic halves of TM10 and TM11 are rotated by 31 and 26 degrees, respectively, away from the centre of the transporter (Fig. 3a,b). Similar bending of TM10 and TM11 was also observed in the inward-facing NarK structures (PDB accession code: 4JR9)^19^; however, the TM10 and TM11 helices are further tilted towards the N bundle side in the present crystal structure (Supplementary Fig. 4a,b). The present crystal structures further reveal the structural change in TM7: as compared with the occluded state structure, the cytoplasmic half of TM7 in the inward-open state structure is rotated by 18 degrees towards the periphery, at the conserved G268 residue in the inward-open structure (Fig. 3a,b). As TM7 is located adjacent to TM10 and TM11 and forms tight interactions (Fig. 2a,b), the structural change of TM7 may be coupled with those of TM10 and TM11. G268 is strictly conserved among the NNP family transporters, including NarU from *E. coli* and NRT2.1 from *A. thaliana* (Supplementary Fig. 3), suggesting that a similar bending of TM7 occurs in other NNP transporters.

To confirm the importance of the conserved Gly residues in TM7, TM10 and TM11, we performed a genetic analysis of NarK mutants of these Gly residues. In this analysis, we examined the complementation activity of NarK mutants introduced by a plasmid, using a mutant *E. coli* strain lacking the operons of all nitrate and nitrite transporters, as well as two of the three nitrate reductases (BW25113 Δ*nirC* Δ*narZ-narU* Δ*narK* Δ*napA-napG-napH-napB* (DE3))^15^. Using the ‘no-induction system', the complementation activities of NarK mutants were visualized by the colour of *E. coli* spots on MacConkey-glucose-nitrate-TMAO (trimethylamine N-oxide) agar plates under anaerobic conditions^15^ (see Methods). The results showed that the G408A and G418A mutants of TM11 and the triple Ala mutant of G363, G365 and G367 in TM10 (ΔG3 mutant) completely abolished the nitrate-uptake activity (Fig. 3c). These results suggested the importance of the flexibility of TM10 and TM11 conferred by these Gly residues. Furthermore, the G268A mutant of TM7 also completely abolished the nitrate-uptake activity (Fig. 3c), suggesting that the bending of TM7 around the G268 residue is critical for the nitrate transport activity by NarK.

Although no structural difference was observed in the other TM helices (that is, TM2, TM4, TM5 and TM8), they also include many conserved Gly residues (Supplementary Fig. 3). Especially, G164, G165, G168, G171, G172, G174 and G177 in TM5 are included in Nitrate Signature motif 1 (refs 25, 26). To investigate the importance of these Gly residues, we performed the genetic analysis of their mutants. The results also indicated the importance of the Gly residues, G85 in TM2, G141 and G144 in TM4, G164, G168, G172 and G177 in TM5, and G308 and G309 in TM8, for the nitrate-uptake activity (Supplementary Fig. 5).

### Structural change in the transport path

**Figure 4:**
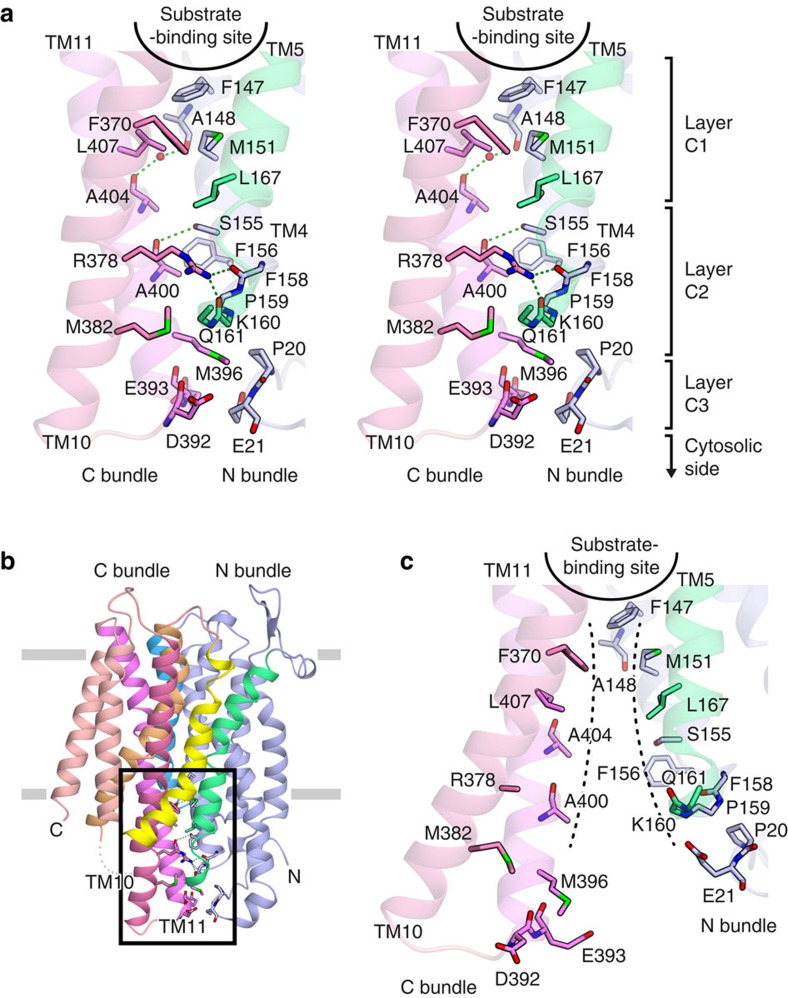
The structural change in the cytosolic transport pathway of NarK. (**a**) The cytoplasmic interactions between the N and C bundles of the occluded state (stereo view). Residues related to the interactions are depicted with stick models. The side chains from F158 to Q161 are omitted for clarification. Water molecules are represented by red spheres. The green dashed lines indicate hydrogen bonds. The area highlighted in this panel is indicated by the rectangle in the overall structure shown in **b**. (**b**) The overall structure of NarK in the nitrate-bound occluded state. The side chains involved in the cytoplasmic interactions are depicted by stick models. (**c**) The cytoplasmic area between the N and C bundles of the inward-open state. The black dashed lines indicate the transport pathway observed in the inward-open state, which is closed in the occluded state.

In the crystal structure of the nitrate-bound occluded state, both the cytosolic and periplasmic sides are tightly closed by hydrophilic and hydrophobic interactions between the N and C bundles (Fig. 4a and Supplementary Fig. 6a,b), and many of these interactions were not observed in the NarU structure previously reported as the occluded state (PDB accession code: 4IU8, Mol A)^18^ (Supplementary Fig. 4c,d). Furthermore, the present high-resolution crystal structures reveal the atomic details of these interactions, including water-mediated interactions (Supplementary Fig. 4c,d and Supplementary Fig. 6b). In the cytoplasmic pathway, F370 and L407 in the C bundle hydrophobically interact with F147, M151 and L167 in the N bundle, forming the interaction layer C1 (Fig. 4a). Furthermore, the main-chain carbonyl groups of A404 in TM11 and A148 in TM4 form a water-mediated hydrogen bond (Fig. 4a). These interactions in layer C1 occlude the pathway from the substrate-binding site towards the cytosolic side. In addition to these hydrophobic interactions, TM4 and TM5 in the N bundle form a number of hydrophobic and hydrophilic interactions with TM10 and TM11 in the C bundle, thereby closing the transport pathway (layers C2 and C3; Fig. 4a). In layer C2, the side chain of F156 in TM4 and the main chain of P159, K160 and Q161, in the loop between TM4 and TM5, form hydrophobic interactions with the side chains of M382 in TM10 and M396 and A400 in TM11 (Fig. 4a). The guanidinium group of R378 in TM10 hydrogen bonds with the main-chain carbonyl groups of F158 and K160, and the main-chain carbonyl group of A400 hydrogen bonds with the hydroxyl group of S155 in TM4 (Fig. 4a and Supplementary Fig. 7a). In layer C3, part of the N-terminal loop consisting of P20 and E21 forms van der Waals contacts with the cytosolic terminus of TM11, including D392, E393 and M396 (Fig. 4a). These interactions between the N and C bundles tightly close the pathway to the cytoplasmic side to prevent the intrusion of water or other solvent molecules into the substrate-binding site. In the structure of NarU, the cytoplasmic pathway is reportedly closed by a thin gate consisting of only two residues, F145 and F367 (corresponding to F147 and F370 in NarK, respectively), and the extensive interactions involving TM4 and TM5 in the N bundle and TM10 and TM11 in the C bundle are missing^18^ (Supplementary Fig. 4d). However, given that several residues of NarK involved in the above interactions, including R378, are conserved in NarU (Supplementary Fig. 3), the cytoplasmic side of NarU would be further closed by the interactions involving TM4, TM5, TM10 and TM11 in the occluded state. Therefore, the previously reported occluded state structure of NarU^18^ may actually represent the partially occluded inward-facing state (Supplementary Fig. 4a,b).

In contrast to their conformations in the present occluded structure of NarK, in the inward-open structure, TM7, TM10 and TM11 in the C bundle are bent at the conserved Gly residues (Fig. 3b). As a result, none of the interactions in layers C1, C2 and C3 is formed (Fig. 4b). The transport pathway is thereby formed between the N and C bundles, and the substrate-binding site is accessible from the cytosolic side (Fig. 4b). The previous structure of NarK bound to the antibody Fab fragment is reported in the inward-facing conformation (PDB accession code: 4JR9)^19^. However, the hydrophobic interactions between F147, M151 and L407 are still formed, and thus the substrate-binding site is not accessible in the previous structure (Supplementary Fig. 4a,b). Therefore, the previously reported structure of NarK is still in the intermediate, partially occluded inward-facing state. In contrast, TM10 and TM11 in the present inward-open structure are further rotated towards the periphery, thereby completely opening the cytoplasmic pathway (Fig. 4c and Supplementary Fig. 4a,b).

### Nitrate recognition in the substrate-binding site

**Figure 5:**
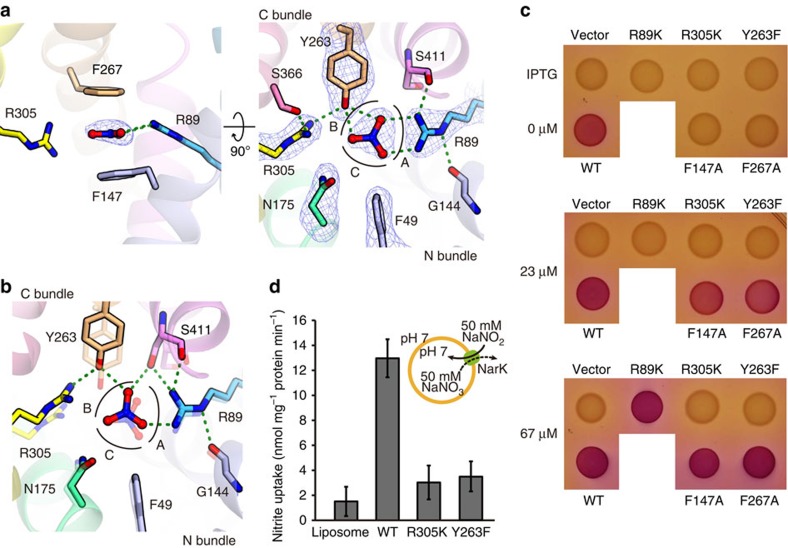
The nitrate recognition mechanism and the structural change in the substrate-binding site of NarK. (**a**) Close-up views of the substrate-binding site in the nitrate-bound occluded state (Mol A), viewed from two different directions. The electron-density map of the *mF*_o_*–DF*_c_ omit maps, contoured at 4*σ* for nitrate ion (left panel) and nitrate ion, F49, R89, N175, Y263 and R305 (right panel), respectively. The colours of the carbon atoms represent the TM helices with the same colour scheme as in Fig. 2a,b. The green dashed lines indicate the hydrogen bonds up to 3.1 Å. (**b**) Close-up view of the substrate-binding site in the nitrate-bound inward-open state. The residues in the occluded state are semitransparent, for comparison. (**c**) Genetic analysis of the nitrate transport activity of NarK mutants directly involved in substrate recognition, using the inducible system. The results are summarized in the table on the right. (**d**) Liposome-based nitrite-flux assay of NarK mutants (Y263F and R305K). All error bars represent the s.d. of three independent trials.

In the nitrate-bound occluded structure, we observed a strong electron-density peak in the substrate-binding site (Fig. 5a). This flat, triangular density peak was present in a location consistent with those of the bound nitrate and nitrite ions in the NarU^18^ and NarK^18^ structures, respectively. The crystallization conditions for the occluded-state crystals (Crystal III) contained ammonium nitrate. Therefore, we assigned this peak as a nitrate ion. The present crystal structures have clarified the interactions between the substrate and the protein side chains (Fig. 5a), as compared with the previous crystal structures of NarU^18^ and NarK^18^. The substrate-binding site is mainly formed by the side chains of F49, R89, F147, N175, Y263, F267 and R305 (Fig. 5a and Supplementary Fig. 8a,b), which are highly conserved among the NNP family transporters (Supplementary Fig. 3). The nitrate ion is sandwiched between F147 and F267, and forms a *π–π* stacking interaction with the *π* electrons of their phenyl groups (Fig. 5a and Supplementary Fig. 8a,b). The oxygen atoms of the nitrate ion are further recognized by three sites: A, B and C (Fig. 5a and Supplementary Fig. 8a,b). In Site A, two oxygen atoms of the nitrate ion form a bidentate salt bridge with the NH1 and NH2 atoms of the R89 guanidinium group. This guanidinium group of R89 is anchored by the main-chain carbonyl groups of G144 in TM4 and S411 in TM11, and together they form a rigid substrate-binding site (Fig. 5a and Supplementary Fig. 8a,b). In Site B, two oxygen atoms of the nitrate ion form a bifurcated hydrogen bond with the phenol hydroxyl group of Y263 in TM7. This hydroxyl group hydrogen bonds with the R305 guanidinium group, which in turn hydrogen bonds with the S366 hydroxyl group (Fig. 5a and Supplementary Fig. 8a). As a result, the guanidinium group of R305 is oriented perpendicularly to the substrate nitrate ion. In Site C, the side-chain amide group of N175 and the phenyl group of F49 form an interaction site for the nitrate ion (Fig. 5a and Supplementary Fig. 8a,b). Overall, these conserved amino-acid residues create a suitable substrate-binding site for a flat anion with delocalized *π* electrons.

Similar nitrate/nitrite recognition by Arg side chain and/or aromatic groups is also observed in several proteins. For example, in molybdenum-containing NAD(P)H:nitrate reductase, the nitrate ion is reportedly recognized by two Arg residues^27^. In contrast, the manner of nitrate recognition by the other nitrate transporter, NRT1.1, is quite different from that of NarK, as NRT1.1 recognizes nitrate by Thr and His residues^21^,^22^.

In the nitrate-bound, inward-open structure, a triangular electron-density peak of a nitrate ion was also observed in the substrate-binding site (Supplementary Fig. 7b). However, we found several differences in the recognition manner of the substrate nitrate, as compared with that in the occluded state. In the inward-open structure, the bending of TM7, TM10 and TM11 leads to a slight movement of the side chains of Y263 in TM7 and R305 in TM8, away from the centre of the transporter (Fig. 5b). As a result, the following changes were observed in the interactions in Sites A and B. In Site A, the distance between one oxygen atom of the nitrate ion and the R89 guanidinium group is increased from 2.7 to 3.1 Å (Fig. 5b and Supplementary Fig. 8a,c). In Site B, the distance between the nitrate ion and the Y263 phenol group is increased from 2.4 to 3.9 Å, and only one oxygen atom is within hydrogen-bonding distance with the Y263 phenol group (Fig. 5b and Supplementary Fig. 8a,c). These changes in Sites A and B increase the volume of the substrate-binding site in the inward-open state structure (37.6 and 50.5 Å^3^ for the occluded and inward-open structures, respectively; Supplementary Fig. 9a,b). Taken together, the overall structural change in the transition from the nitrate-bound occluded to the inward-open state, including the bending of TM7, TM10 and TM11, induces the movement of Y263 and R305 in Site B, which enlarges the volume of the substrate-binding site. This change in the substrate-binding site may result in decreased substrate affinity in the inward-open state, thereby facilitating substrate release towards the cytoplasmic side.

In contrast, the *apo* inward-open structure was similar to the nitrate-bound, inward-open structure (r.m.s.d. of 0.51 Å for 435 Cα atoms; Supplementary Fig. 8c,d and Supplementary Fig. 10a,b). While no electron-density peak was observed in the substrate-binding site in the *apo* structure of the inward-open state (Supplementary Fig. 7c), the positions of the side chain atoms in the substrate-binding site are superimposable on those of the nitrate-bound structure (Supplementary Fig. 10b). This suggested that the inward-open state is also stable without the substrate, and that the binding and release of the substrate can occur without any large structural changes.

### Mutational analysis of the substrate recognition residues

To examine the importance of the residues involved in the substrate recognition, we performed the structure-based genetic analysis of NarK mutants using the ‘inducible system' (see Methods). The results showed that the Ala mutants of both F147 and F267, which form *π–π* interactions with nitrate (Fig. 5a), did not restore the nitrate-transport activity to the same level as that of wild-type NarK (Fig. 5c), while complementation was observed with higher isopropyl-β-D-thiogalactoside (IPTG) concentrations (23 and 67 μM; Fig. 5c). This result suggested that the *π–π* stacking interaction between the substrate nitrate and the phenyl groups of F147 and F267 is important, but not critical for the transport activity. The R89K mutant also abolished the complementation activity, but rescued the nitrate-uptake activity under the conditions with the highest IPTG concentration (67 μM; Fig. 5c). This result suggested that the positive charge at the R89 position is important for the substrate recognition. In contrast, the Y263F and R305K mutants completely abolished the complementation activity, even under the conditions with the higher IPTG concentrations (23 and 67 μM; Fig. 5c). The results obtained with the Y263F mutant suggested that the phenol hydroxyl group of Y263, which hydrogen bonds with both the substrate nitrate and R305 guanidinium group, is critical for the transport activity. In addition, the results from the R305K mutant suggested that the guanidinium group of R305 itself, rather than simply a positive charge, is essential for the transport activity. Furthermore, the purified Y263F and R305K mutants showed no transport activity in our reconstituted system (Fig. 5d). The results from these genetic and *in vitro* analyses support our proposal that Y263 and R305 in the substrate-binding site are the key residues for coupling the bending of the TM helices with the substrate recognition.

### MD simulation

**Figure 6:**
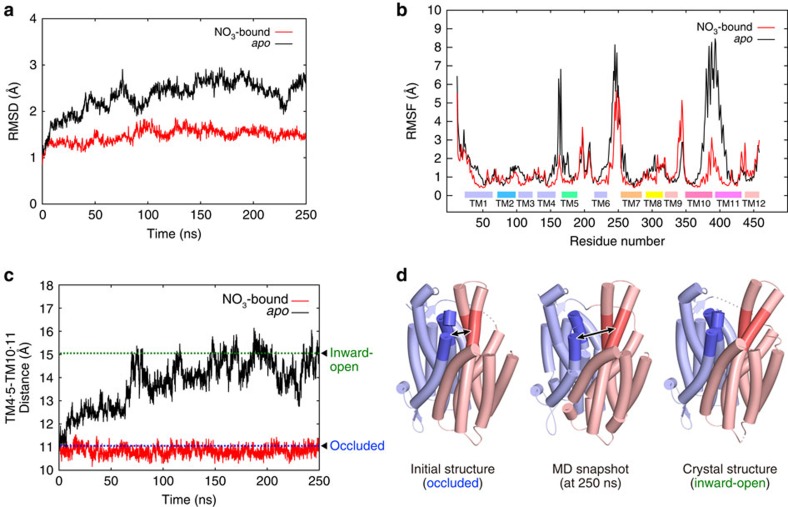
Molecular dynamics simulations of NarK. (**a**,**b**) Structural deviations and fluctuations observed in the molecular dynamics simulations. (**a**) Plots of the RMS deviations of the overall Cα atoms from the initial crystal structures as a function of time, during the nitrate-bound and *apo* occluded simulations. (**b**) Plots of the time-averaged RMS fluctuations of each residue from the initial crystal structures, during the nitrate-bound and *apo* occluded simulations. The locations of the TM segments are indicated by the boxes, which are coloured as in Fig. 2a,b. (**c**) Plots of the distances between the centroids of the Cα atoms of the cytosolic parts of TM4-TM5 (A148 to G171) and TM10-TM11 (T369 to L380 and A399 to F409), as a function of time, during the nitrate-bound and *apo* occluded simulations. The distances between the centroids of the Cα atoms of the cytosolic parts of TM4-TM5 and TM10-TM11 of two crystal structures (occluded and inward-open state) are shown by blue and green dashed lines, respectively, and indicated by black rectangles. (**d**) The initial structure, the snapshot structure of the *apo* simulation at 250 ns, and the crystal structure in the inward-open state are shown. The N and C bundles are coloured pale blue and pink, respectively. The regions in TM4, TM5, TM10 and TM11 used for the distance measurement are highlighted in deep blue and red colours and are indicated by black arrows.

To clarify the dynamic coupling mechanism between the state transition and the substrate recognition, we performed MD simulations based on the present crystal structure. In each of three independent simulations starting from the occluded state with the bound nitrate, no large structural change in the overall conformation was observed during the 250-ns simulation (Fig. 6a and Supplementary Fig. 11a,b), suggesting that the overall structure of the occluded state is stable in the lipid bilayer environment. The structure of the substrate-binding site, including the nitrate recognition manner, remained unchanged during the simulation (Supplementary Fig. 12a), showing that the substrate-binding site of the occluded state structure is stable with the bound nitrate.

Next, to examine the effect of the nitrate-binding and release on the occluded state structure, we performed the three independent simulations starting from the occluded state without the bound nitrate. Interestingly, a large structural change was observed in the first 10 ns (Fig. 6a), mainly in the TM10 and TM11 helices in the C bundle (Fig. 6b). The interaction between TM4–TM5 and TM10–TM11 was lost in the first 10 ns (Fig. 6c), including the hydrogen bonds between R378 and the loop connecting TM4 and TM5 (Supplementary Fig. 12b). Similar structural changes were reproducibly observed in the other two simulations (Supplementary Fig. 11c,d). The transport path from the cytosolic side to the substrate-binding site opened, and water molecules entered the substrate-binding site (Supplementary Fig. 13a,b). After 250 ns in the simulation, the distance between TM4–TM5 and TM10–TM11 was increased to a similar level as that in the inward-open structure (Fig. 6c,d). In addition, we also performed the MD simulation starting from the *apo* inward-open structure. The result showed that the *apo* inward-open structure is stable, and no structural change towards the occluded state was observed without any substrates during the 250-ns simulation (Supplementary Fig. 14).

Taken together, these results suggested that the negatively charged nitrate ion, bound between the positively charged R89 and R305 residues, relaxes the electrostatic repulsion between them, thereby facilitating the formation of the occluded state. In contrast, without the substrate nitrate ion, the electrostatic repulsion between R89 and R305 allows the N and C bundles to segregate from each other, thereby facilitating the formation of the inward-open state.

## Discussion

The locations of the nitrate ions found in the present NarK structures are consistent with those of the nitrite ion in the previous crystal structure of NarK^19^, suggesting that both nitrate and nitrite bind to the same substrate-binding site competitively. The present atomic-resolution structures of the nitrate-bound NarK provided further structural insights into the nitrite ion recognition mechanism (Supplementary Fig. 15a). Given that nitrite also has a negative charge and delocalized *π* electrons, it can bind to the substrate-binding site between the phenyl groups of F147 and F267, as observed in the nitrate-bound structures. The interactions in Sites A and B are also possible, in the case of nitrite. The nitrite oxygen atoms can form a bidentate salt bridge with the NH1 and NH2 atoms of R89, and hydrogen bond with the hydroxyl group of Y263 (Supplementary Fig. 15a), as in the nitrate-bound structures (Fig. 5a). The nitrogen atom of nitrite has lone-pair electrons, and thus it could further hydrogen bond with the hydroxyl group of Y263 (Supplementary Fig. 15a). In contrast, nitrite has only two oxygen atoms, and is smaller than nitrate. Therefore, the interactions involving one of the three oxygen atoms of nitrate are impossible in the case of nitrite. In our nitrite-bound model, the side-chain amide group of N175 (that is, Site C) is not involved in substrate recognition (Supplementary Fig. 15a). This notion is consistent with the results from a previous study, showing that the affinity of NarU for nitrite is ∼10-fold lower than that for nitrate^18^.

The present *in vitro* functional analysis revealed that chloride ions weakly facilitate the nitrate-uptake activity (Fig. 1b), suggesting that the chloride ion binds to the substrate-binding site to be transported as a counteranion. Although the chloride ion lacks *π* electrons, it can bind between the R89 and R305 residues to induce the formation of the occluded state. This is consistent with the results of the genetic analysis of F147 and F267, showing that the *π–π* stacking interaction with the substrate is important, but not critical for the transport activity. The results of the *in vitro* functional analysis also showed that carbonate and formate ions weakly facilitate the nitrate-uptake activity (Fig. 1b). Given that carbonate ion is singly protonated to form bicarbonate ion under the assay conditions, the proton of the bicarbonate cannot fit within the substrate-binding site (Supplementary Fig. 15b). This may reduce the affinity of the bicarbonate ion to the substrate-binding site, as compared with that of the nitrate ion, despite their similar molecular shapes. The formate ion has a hydrogen atom bound to the carbon atom, while the nitrite ion has lone pair electrons at the corresponding nitrogen atom. Thus, the formate ion also cannot fit within the substrate-binding site, although it has a similar molecular shape as the nitrite ion (Supplementary Fig. 15c). In contrast, the present *in vitro* analysis showed that acetate and propionate ions do not facilitate the nitrate-uptake activity (Fig. 1b). The bulky methyl and ethyl groups may completely prevent the interaction of these anions with the substrate-binding site, and thus they are not transported as counteranions. These results with the acetate and propionate ions also suggested that NarK is a tightly coupled antiporter, which does not transport substrates in the absence of counteranions.

**Figure 7:**
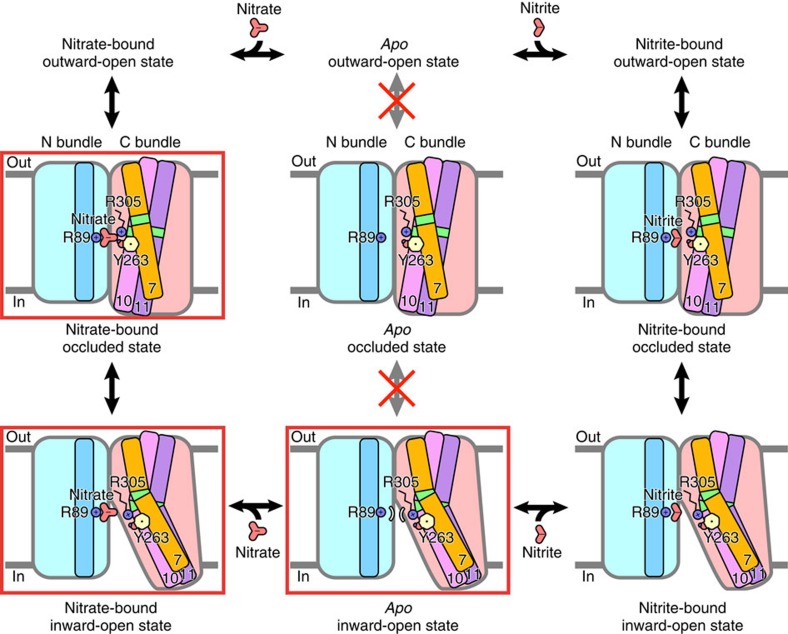
Working model of nitrate/nitrite antiport by NarK. The key residues in the coupling between the structural change and the substrate recognition (R89, Y263 and R305) and substrates (nitrate and nitrite) are shown. The TM helices that become bent during the transport cycle (TM7, TM10 and TM11) in the C bundle are illustrated as amber, pink and purple sticks, respectively, while those in the N bundle are represented by light blue sticks. The approximate locations of the important Gly residues for the TM bending are coloured green. The states corresponding to the present crystal structures are highlighted by red rectangles.

In conclusion, we demonstrated that NarK is a nitrate/nitrite antiporter, by an *in vitro* functional analysis. This conclusion is also consistent with the results of MD simulations with and without nitrate in the substrate-binding site. Under physiological conditions, it is reasonable that NarK functions as a system for both the uptake of nitrate, as an electron acceptor for anaerobic respiration and the extrusion of cytotoxic nitrite produced by anaerobic respiration. The present crystal structures and structure-based genetic analyses have elegantly explained the coupling mechanism between the substrate recognition and the structural changes that underlie the nitrate/nitrite antiport mechanism. Our working model of nitrate/nitrite antiport by NarK explains the half of the transport cycle involving the inward-open state. In the nitrate-bound occluded state, the negative charge of the nitrate in the substrate-binding site relaxes the electrostatic repulsion between the two positively charged arginine residues (R89 and R305), thereby enabling these two arginine residues to be in close proximity in the low-dielectric intramembrane environment (Fig. 7). The following structural change to the inward-open state induces the bending of the helices, TM7, TM10 and TM11, around the conserved Gly residues, which opens up the transport pathway from the substrate-binding site to the cytoplasmic side between the N and C bundles (Fig. 7 and Supplementary Movie). These bending motions of the TM helices are coupled with the movement of the side chains of Y263 in TM7 and R305 in TM8 away from the N-bundle side, which enlarges the volume of the substrate-binding site and decreases the affinity of NarK for nitrate, thereby facilitating nitrate release to the cytoplasm. The key residues for this coupling mechanism (Y263, R305 and G268) are strictly conserved in the NNP family (Supplementary Fig. 3), suggesting that other NNP family members, including NRT2.1 of *A. thaliana*, transport nitrate ions in a similar manner. The electrostatic repulsion between the positive charges of R89 and R305 prohibits the movement of Y263 and R305 towards the N-bundle side, thus preventing the closure of the transport pathway by the movement of TM7, TM10 and TM11 of the C bundle towards the N-bundle side (Fig. 7 and Supplementary Movie). In the next step, a nitrite ion in the cytoplasm is bound to the substrate-binding site in a similar manner to that of nitrate (Supplementary Fig. 15a), which may facilitate the conformational change to the outward-open state via the nitrite-bound occluded state. In this structural change between the occluded and outward-open states, the conserved Gly residues in TM2, TM4, TM5 and TM8 may play important roles in the conformational flexibility, as suggested by our genetic analysis (Supplementary Fig. 5). Further understanding of the remaining half of the transport cycle awaits the determination of the outward-open structure at atomic resolutions.

## Methods

### Expression and purification of NarK

The *narK* gene (GI: 945783) was cloned from the *E. coli* str. K-12 substr. MG1655 genome (ATCC 47076) using the NarK (WT) primers described in Supplementary Table 1, and subcloned into a pET-modified vector^28^. The resultant plasmids were expressed in *E. coli* C41(DE3) Δ*acrB* cells, and the proteins were purified as follows. The cells were grown in a 10-l culture at 37 °C to an absorbance at 600 nm of 0.4–0.6, and expression was induced with 0.5 mM IPTG at 20 °C for 24 h. The cells were then harvested using centrifugation (4,500*g*, 10 min, 4 °C), resuspended in buffer containing 50 mM Tris-HCl (pH 8.0), 300 mM NaCl, 20 mM NaNO_3_, 0.1 mM phenylmethylsulfonyl fluoride (PMSF) and 4 mM β-mercaptoethanol (β-ME), and disrupted by three passages through a Microfluidizer processor (Microfluidics) at 15,000 p.s.i. After removal of the debris using centrifugation (12,000*g*, 30 min, 4 °C), the supernatant was ultracentrifuged (138,000*g*, 1 h, 4 °C) to pellet the membranes, which were then solubilized in a buffer containing 50 mM Tris-HCl (pH 8.0), 300 mM NaCl, 20 mM NaNO_3_, 0.1 mM PMSF, 4 mM β-ME, 2% n-dodecyl-β-D-maltoside (DDM) and 0.4% cholesteryl hemisuccinate (CHS). Insoluble materials were removed using ultracentrifugation (138,000*g*, 30 min, 4 °C), and the supernatant was mixed with 10 ml Ni-NTA Superflow resin (Qiagen). After binding for 2 h, the resin was washed with 50 mM Tris-HCl (pH 8.0), 300 mM NaCl, 25 mM NaNO_3_, 4 mM β-ME, 0.05% DDM, 0.01% CHS and 20 mM imidazole, and NarK was eluted with the same buffer supplemented with 300 mM imidazole at 4 °C. The GFP-His_8_ tag was cleaved by a His-tagged tobacco etch virus protease (produced in-house) and dialysed against the buffer without imidazole. The sample was reloaded on the 10-ml Ni-NTA column, to remove the protease. The flow-through fraction containing NarK was collected, concentrated and further purified on a HiLoad 16/600 Superdex 200 pg column (GE Healthcare), in 20 mM Tris-HCl (pH 8.0), 150 mM NaCl, 25 mM NaNO_3_, 4 mM β-ME, 0.05% DDM and 0.01% CHS. The purified protein was concentrated to 15–20 mg ml^−1^ with a centrifugal filter device (Millipore, 50 kDa molecular weight cutoff) and frozen until crystallization.

### Crystallization

For the LCP crystallization trials, the protein solution was mixed with mono-olein (1-oleoyl-rac-glycerol) at a 4:6 w/w ratio using coupled syringe devices, and the 30- to 80-nl protein-laden LCP droplets were overlaid with 1 μl precipitant solution on either 96-well plastic sandwich plates by the crystallization robot, mosquito LCP (TTP LabTech) or glass sandwich plates by the repeating dispenser (Hamilton). The crystals of NarK were grown at 20 °C, and three different types of crystals (I, II and III) were obtained in reservoir solutions I, II and III, respectively (reservoir solution I: 27% PEG500DME, 100 mM MES-NaOH (pH 6.5), 100 mM NH_4_NO_3_; reservoir solution II: 36% PEG400, 100 mM MES-NaOH (pH 6.0), 10 mM Zn(OAc)_2_, 3% sucrose; reservoir solution III: 31% PEG500DME, 100 mM MES-NaOH (pH 5.8), 100 mM NH_4_NO_3_). All crystals grew to full size in 2–3 weeks. The heavy atom-derivative crystals were prepared using the soaking method. After the native crystal III was grown on glass sandwich plates to the full size, ∼15 × 40 × 80 μm, the overlaid crystallization solution (1 μl) was replaced by 1–1.5 μl of the solution supplemented with a slightly lower concentration of PEG500DME and 4 mM CH_3_HgCl. The crystal was incubated at 20 °C for 5 h without drying. It was harvested using micromounts (MiTeGen), and was flash-cooled and stored in liquid nitrogen.

### Data collection and structure determination

Diffraction data were collected at the SPring-8 BL32XU beamline, using a 1 μm × 15 μm (width–height) microbeam^29^. The data-processing statistics are summarized in Table 1. The structure was determined using the single isomorphous replacement with anomalous scattering (SIRAS) method, using the native and CH_3_HgCl-soaked crystals. Six Hg atom sites were identified with the programme SHELXD^30^,^31^. The initial phases were calculated using the programme SHARP^32^, followed by solvent-flattening with SOLOMON^33^ and twofold noncrystallographic symmetry averaged using DM^34^. The initial model structure of NarK was built with the programme Phyre2 (ref. 35), using the glycerol-3-phosphate transporter GlpT structure (PDB accession code: 1PW4)^36^ as a template. The model was further built manually using COOT^37^,^38^ and refined using PHENIX^39^,^40^. Using this Crystal III structure as a search model, the Crystal I and II structures were determined with the molecular replacement method, using the programme PHASER^41^. Crystals I and II contain one molecule in each asymmetric unit, while Crystal III contains two molecules (Mol A and B) in each asymmetric unit. Mol A and B in Crystal III are essentially the same (r.m.s.d. of 0.38 Å for all Cα atoms), and thus we only discussed Mol A in the main text. The structure refinement statistics are summarized in Table 1.

### MD simulations

We performed MD simulations starting from the *apo* occluded, nitrate-bound occluded and *apo* inward-open states. The present crystal structures of the nitrate-bound occluded and *apo* inward-open states were used as initial models. All of the lipid molecules observed in the crystal structures were removed, while all of the water molecules observed in the crystal structures were kept. The disordered loop connecting the N and C bundles was modelled by the programme MODELLER^42^. For the *apo*-occluded simulation, the bound nitrate ion was removed from the crystal structure. The missing side chains and hydrogen atoms were built with the programme VMD^43^. The prepared structures were then embedded in a fully hydrated POPE bilayer^44^. In the crystal structure, several mono-olein molecules were observed around the TM segments, and these were used as indicators for the locations of the membrane. The lipid molecules overlapping the protein were removed, resulting in 320 POPE molecules included overall. The lipid–protein complex was then hydrated to form the 96 Å × 96 Å × 96 Å simulation box. Sodium and chloride ions were then added to neutralize the system with a salt concentration of 150 mM. The molecular topologies and parameters from the CHARMM36 force-field parameters, with *ϕ*, *ψ* cross-term map correction, were used^44^.

MD simulations were performed with the programme NAMD 2.8 (ref. 45). The systems were first energy minimized for 1,000 steps with fixed positions of the nonhydrogen protein atoms, and then for another 1,000 steps with 10 kcal mol^−1^ restraints for the nonhydrogen protein atoms. For the equilibration, the system was first subjected to a 100-ps simulation run with harmonic restraints on the nonhydrogen protein atoms in the NPAT ensemble, to relax the solute molecules, and then was further equilibrated by a 1-ns simulation run with harmonic restraints on the non-hydrogen protein atoms in the NPT ensemble. Finally, 250-ns MD simulations without any restraints were performed in the NPT ensemble for the production runs. In these simulations, constant pressure (1 atm) and temperature (300 K) were maintained using Langevin dynamics and a Langevin piston, respectively. The particle mesh Ewald method was employed for the calculation of the electrostatic interactions^46^. The equation of motion was integrated with a time step of 2 fs.

### Liposome-based nitrite-flux assay

For the preparation of NarK-reconstituted proteoliposomes, soybean lipid (L-α-phosphatidylcholine-type IV-S; Sigma) was dissolved in chloroform and evaporated at room temperature. The lipid film deposited on the interior of a plastic tube was resuspended to a final concentration of 10 mg ml^−1^ in extraliposome buffer, containing 25 mM HEPES-NaOH (pH 7.0), and 50 mM of either NaNO_3_, NaCl, NaHCO_3_, HCOONa, CH_3_COONa or CH_3_CH_2_COONa. The suspension was sonicated for 30 s to form unilamellar vesicles. Purified proteins were reconstituted into liposomes, at a lipid/protein ratio of 100:1 (w/w), by three rounds of freezing and thawing. The proteoliposomes were centrifuged at 210,000*g* for 30 min. The nitrite-uptake reaction was initiated by resuspending the pellet in intraliposome buffer, containing 25 mM HEPES-NaOH (pH 7.0) and 50 mM NaNO_2_. After an incubation at room temperature for 1 h, the reaction mixture was loaded on a Sephadex G-50 column (0.5 × 4.0 cm) equilibrated with 25 mM HEPES-NaOH (pH 7.0). The eluted fraction was mixed with sodium dodecyl sulfate, to disrupt the liposomes, and then with HCl, in order to lower the pH, to final concentrations of 0.5% and 0.1 M, respectively. The solution was mixed with DAF-FM^23^ and incubated at room temperature for 2 min. The solution was diluted 10-fold with 25 mM HEPES-NaOH (pH 7.0) and incubated at room temperature for 2 min. The fluorescence of triazolofluorescein was monitored with excitation at 500 nm and emission detected at 515 nm, using an F-7000 fluorescence spectrophotometer (Hitachi). To generate the membrane potential in proteoliposomes, we added 10 mM KCl to the intraliposome buffer, and 100 mM KCl with 10 μM valinomycin to the extraliposome buffer. To change the pH of the intra- and extraliposome buffers, MES-NaOH (pH 6.0) and Tris-HCl (pH 8.0) were used.

### Genetic analysis of the transport activity of NarK mutants

The genetic analysis of the NarK mutants was performed essentially as described previously^15^. In brief, in the presence of TMAO under anaerobic conditions, *E. coli* assimilates TMAO, which is reduced to the strong base trimethylamine by endogenous TMAO reductases, resulting in the formation of pale yellow spots on MacConley-glucose-TMAO agar plates. In contrast, if nitrate is assimilated by *E. coli* cells during anaerobic growth, it prevents TMAO reduction by out-competing TMAO reductase for electrons from the quinol pool^15^, resulting in the formation of dark red spots. The *E. coli* strain constructed for this assay lacks all of the endogenous nitrate/nitrite transporters and channels (NarK, NarU and NirC). Therefore, the colours of the cells grown anaerobically in the presence of both nitrate and TMAO completely depend on the nitrate-uptake activity of the plasmid-encoded NarK.

To construct the assay strain with the sextuplet gene knockout (Δ*napA-napB*, Δ*narZ-narU*, Δ*nirC*, Δ*narK*), each gene's knockout allele was constructed and transferred step-by-step to the destination strain BW25113, principally by the method described in ref. 47, using appropriate PCR primers (NAP1 and NAP2 for Δ*napA-napB*, NAR1 and NAR2 for Δ*narZ-narU*; Supplementary Table 2) or P1 phage transduction from a systematic *E. coli* knockout library strain (Δ*nirC*, Δ*narK*)^48^. We isolated λ DE3 lysogenic strains, which carries the inducible T7 polymerase system for the gene expression^49^, by spotting λ DE3 lysate together with appropriate helper phage lysate on the host strain. Under the noninduced conditions in the absence of lactose or IPTG, the assay strain expresses a stable amount of T7 polymerase, which is sufficient for the assays of NarK mutants with severe phenotypes (‘no-induction system'; Fig. 3c). In contrast, to evaluate the nitrate transport activity of NarK and its mutants depending on their expression levels, another expression system was constructed. In the presence of pLysS, the T7 polymerase activity is reduced, thus allowing more stringent control of expression from the T7 promoter, depending on the IPTG concentration (‘inducible system'). We used this inducible system under three different conditions (IPTG=0, 23 and 67 μM) for the assays of mutants involved in substrate recognition (Fig. 5c).

Transformants harbouring the WT or mutant *narK* gene derived from the exogenous plasmid were cultured overnight at 37 °C in liquid LB medium, supplemented with appropriate antibiotics, diluted 50-fold in the same medium and then grown to an optical density (OD)_600_ of 0.6 at 37 °C. The refreshed log-phase cultures were spotted on modified MacConkey-glucose-nitrate-TMAO plates^50^, containing 0.5 g KNO_3_, 0.8 g D-glucose and 2.0 g TMAO in 200 ml MacConkey agar medium, and grown anaerobically at 30 °C for 12 h using AnaeroPack jar systems (AnaeroPack Kenki 5% A-07, Mitsubishi Gas Chemical Company Inc., Tokyo, Japan).

## Author contributions

M.F. expressed, purified and crystallized NarK, and collected the diffraction data and solved the structures. M.F. made the mutants for genetic analyses of the nitrate transport activity of NarK. H.T. and M.F. performed *in vitro* assays using proteoliposomes. K.I. constructed the gene knockout *E. coli* strains and performed genetic analyses. S.D. purified proteins for *in vitro* assays. R.I. performed MD simulations. A.D.M. performed experiments using fluorescent probes before the liposome-based nitrite-flux assay. M.F., H.E.K., K.I., R.I. and O.N. designed the research; M.F., H.E.K., R.I. and O.N. wrote the manuscript. R.I. and O.N. directed and supervised all of the research.

## Additional information

**Accession codes.** The coordinates and the structure factors have been deposited in the Protein Data Bank (PDB), under the accession codes 4U4V (*apo* inward-open state), 4U4T (nitrate-bound inward-open state) and 4U4W (nitrate-bound occluded state).

**How to cite this article:** Fukuda, M. *et al*. Structural basis for dynamic mechanism of nitrate/nitrite antiport by NarK. *Nat. Commun*. 6:7097 doi: 10.1038/ncomms8097 (2015).

## Supplementary Material

Supplementary Figures and Supplementary Tables Supplementary Figures 1-15, Supplementary Tables 1-2

Supplementary Movie 1 Conformational transitions of NarK from the nitrate-bound occluded state toward the nitrite-bound occluded state, via the inward-open state, are shown.

## Figures and Tables

**Table 1:** Data collection and refinement statistics.

	*Apo*, inward-open (Crystal I)	Nitrate-bound, inward-open (Crystal II)	Nitrate-bound, occluded (Crystal III)	CH_3_HgCl derivative
*Data collection*				
X-ray source	SPring-8 BL32XU			
Wavelength (Å)	1.0000	1.0000	1.0000	1.0000
Space group	*P*2_1_2_1_2_1_	*P*2_1_2_1_2_1_	*P*2_1_2_1_2_1_	*P*2_1_2_1_2_1_
Cell dimensions				
*a*, *b*, *c* (Å)	35.5, 106.5, 124.6	35.4, 109.4, 126.0	68.8, 86.1, 221.6	68.8, 86.3, 222.4
*α*, *β*, *γ* (°)	90, 90, 90	90, 90, 90	90, 90, 90	90, 90, 90
Resolution (Å)	50.0–2.35 (2.49–2.35)*	50.0–2.4 (2.54–2.4)	50.0–2.4 (2.54–2.4)	50.0–3.4 (3.52–3.4)
*R*_sym_	0.147 (1.391)	0.243 (1.600)	0.165 (1.111)	0.445 (1.516)
*I*/*σ*(*I*)	7.33 (1.12)	6.79 (1.08)	7.00 (1.10)	10.0 (2.3)
Completeness (%)	88.3 (87.7)	99.8 (99.0)	97.2 (98.6)	95.7 (100.0)
Redundancy	5.2 (4.6)	6.4 (6.3)	4.2 (4.1)	9.0 (9.3)
CC_1/2_	0.996 (0.598)	0.994 (0.477)	0.993 (0.601)	0.989 (0.515)
				
*Refinement*				
Resolution (Å)	50.0–2.35	50.0–2.4	50.0–2.4	
No. of reflections	18,217	19,921	50,895	
*R*_work_/*R*_free_	0.2037/0.2318	0.2170/0.2475	0.2191/0.2368	
No. of atoms				
Protein	3,311	3,292	6,486	
Ligand/ion/lipid	40	77	224	
Water	26	31	104	
*B*-factors				
Protein	63.1	45.1	52.7	
Ligand/ion/lipid	63.2	45.1	52.8	
Water	49.1	37.7	47.1	
R.m.s. deviations				
Bond length (Å)	0.003	0.009	0.002	
Bond angle (°)	0.736	1.169	0.671	
Ramachandran plot				
Favoured (%)	98.17	97.95	97.90	
Allowed (%)	1.83	2.05	2.10	
Outliers (%)	0	0	0	

*Values in parentheses are for highest-resolution shell.
