# Supplementary material for: Structural basis for dynamic mechanism of nitrate/nitrite antiport by NarK
Source: Nat Commun. 2015 May 11;6:7097. doi: 10.1038/ncomms8097 (PMC4432589; doi:10.1038/ncomms8097)
Supplement: Supplementary Figures and Supplementary Tables — Supplementary Figures 1-15, Supplementary Tables 1-2 [file ncomms8097-s1.pdf]

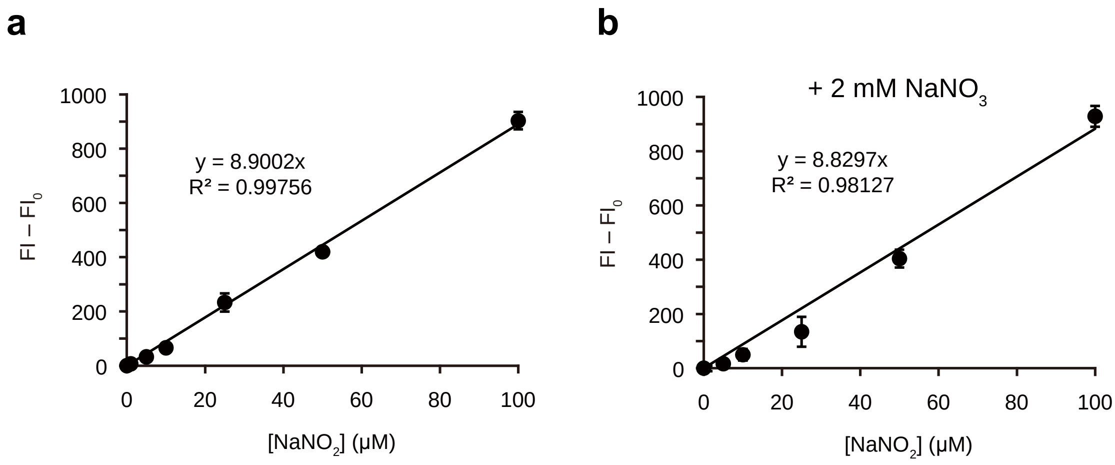

Supplementary Figure 1 Standard curve of DAF-FM as a nitrite-specific probe.

(**a**) Relationship between the nitrite ion concentration and the fluorescence intensity of triazolofluorescein, a nitrite-adduct of DAF-FM. (**b**) The presence of nitrate does not affect the triazolofluorescein formation by nitrite and DAF-FM. FI is the fluorescence intensity and FI_0 is the FI with 0 μM $NaNO_2$. All error bars represent the standard deviation (s.d.) of three independent trials.

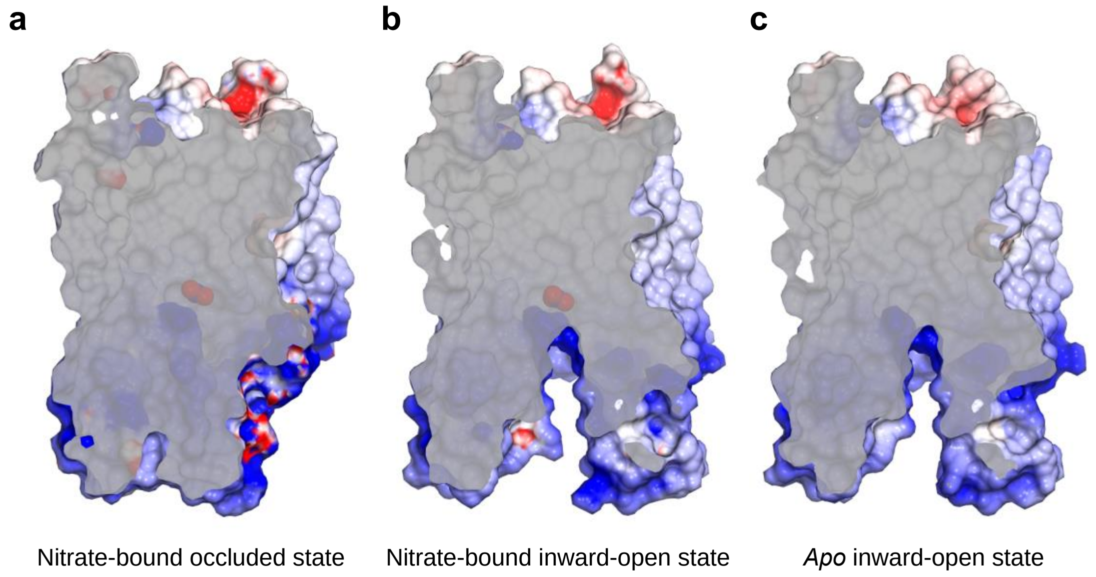

Supplementary Figure 2 Cross-sections of the crystal structures of NarK.

(**a**), (**b**), and (**c**) Cross-sections of the crystal structures of NarK, colored semi-transparent gray, showing the solvent accessible surface calculated with a 1.4 Å radius sphere (assuming a water molecule). The molecular surfaces are colored according to the electrostatic potential, ranging from blue (positive) to red (negative). Bound nitrate molecules are shown as CPK models. (a) Nitrate-bound occluded state (Mol A). (b) Nitrate-bound inward-open state. (c) *Apo* inward-open state.

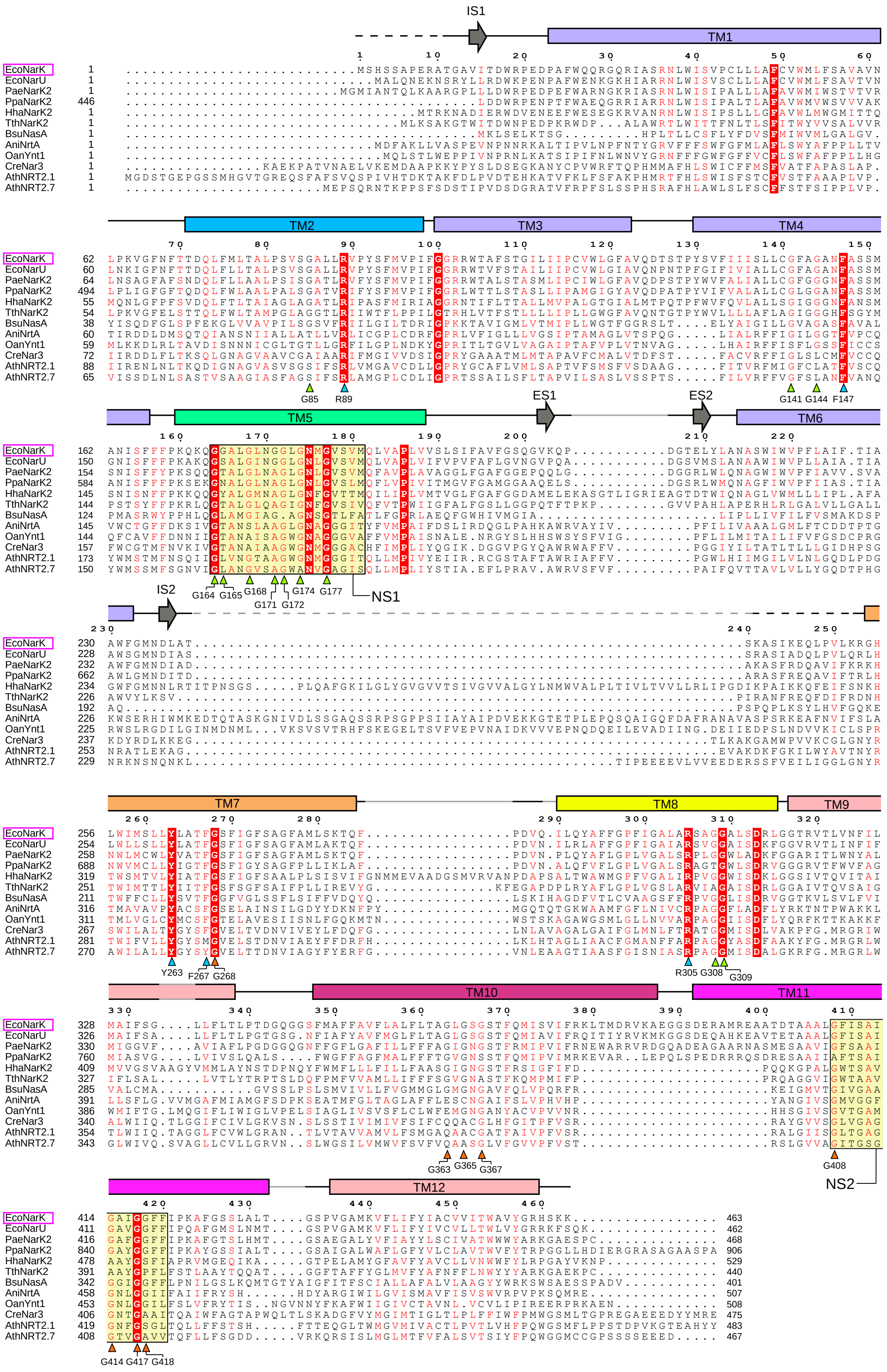

IS1
TM1
TM2
TM3
TM4
TM5
ES1
ES2
TM6
IS2
NS1
TM7
TM8
TM9
TM10
TM11
TM12
NS2
EcoNarK
EcoNarU
PaeNarK2
PpaNarK2
HhaNarK2
TthNarK2
BsuNasA
AniNrtA
OanYnt1
CreNar3
AthNRT2.1
AthNRT2.7
G85
R89
G141 G144
F147
G164 G165
G168
G171 G172
G174
G177
Y263
F267 G268
R305 G308
G309
G363
G365
G367
G408
G414 G417 G418

Supplementary Figure 3 Multiple amino acid sequence alignment of NNP family proteins.

Sequence alignment of *Escherichia coli* NarK (EcoNarK, GI:16129186), *Escherichia coli* NarU (EcoNarU, GI:16129428), *Pseudomonas aeruginosa* PAO1 NarK2 (PaeNarK2, GI:15599071), *Paracoccus pantotrophus* NarK2 (PpaNarK2, GI:14348603), *Halomonas halodenitrificans* NarK2 (HhaNarK2, GI:18413617), *Thermus thermophilus* HB8 NarK2 (TthNarK2, GI:6689413), *Bacillus subtilis* subsp. subtilis str. 168 NasA (BsuNasA, GI:255767099), *Aspergillus nidulans* FGSC A4 NrtA (AniNrtA, GI:67517614), *Ogataea angusta* Ynt1 (OanYnt1, GI:3849986), *Chlamydomonas reinhardtii* Nar3 (CreNar3, GI:437765), *Arabidopsis thaliana* NRT2.1 (AthNRT2.1, GI:15223123) and *Arabidopsis thaliana* NRT2.7 (AthNRT2.7, GI: 15241440). The secondary structure of EcoNarK, determined by the present crystal structures, is indicated above the sequences. The colors of the TM helices correspond to those in Fig. 2, (a) and (b). The α-helices (TM1-12, as described in the main text) and β-strands (IS1-2 and ES1-2, representing the intracellular and extracellular strands, respectively) are indicated by cylinders and arrows, respectively. Strictly conserved residues among the twelve molecules are highlighted in red boxes, and highly conserved residues are indicated by red letters. The conserved Gly residues in TM7, 10 and 11 are indicated by orange triangles. The conserved Gly residues in TM2, 4, 5 and 8 that were mutated for the *in vivo* nitrate uptake assay are indicated by light green triangles. The residues involved in substrate recognition that were mutated for the *in vivo* nitrate uptake assay are indicated by light blue triangles. NS1 and NS2 are representing the Nitrate Signature motif 1 and 2, respectively.

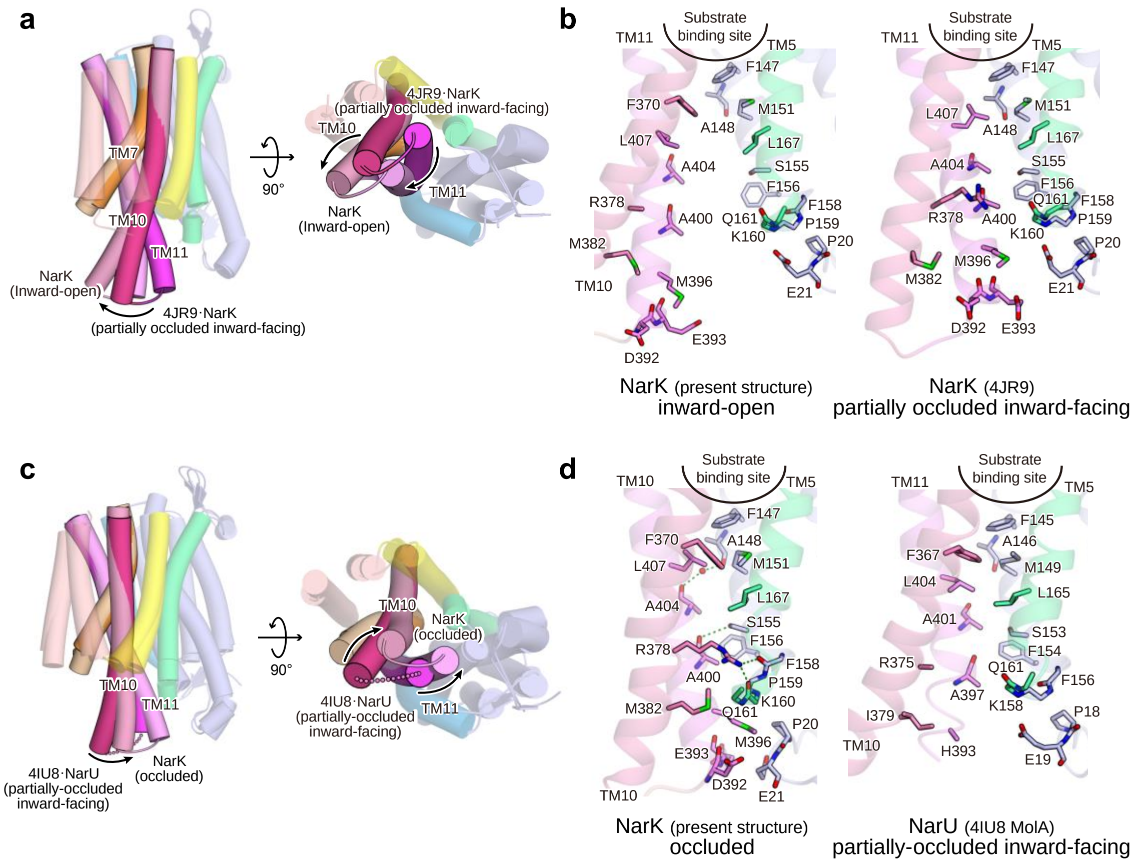

Supplementary Figure 4 Comparison of the present and previously reported structures of nitrate transporters.

(**a**) and (**b**) Structural comparison of the inward-open states of NarK in the present crystal structure and the previously reported structure (PDB accession code: 4JR9). (a) Superimposed structures, viewed from the plane of the membrane (left panel) and the cytoplasmic side (right panel). (b) The cytosolic transport pathway of NarK in the inward-open states in the present crystal structure, left panel, and the previously reported structure (4JR9, right panel). (**c**) and (**d**) Structural comparison between NarK in the nitrate-bound occluded state (Mol A, present crystal structure) and NarU in the nitrate-bound partially-occluded inward-facing state (PDB accession code: 4IU8, Mol A). (c) Superimposed structures, viewed from the plane of the membrane (left panel) and the cytoplasmic side (right panel). (d) The cytosolic transport pathway of NarK in the nitrate-bound occluded state (present crystal structure, left panel) and NarU in the nitrate-bound partially-occluded inward-facing state (4IU8, right panel).

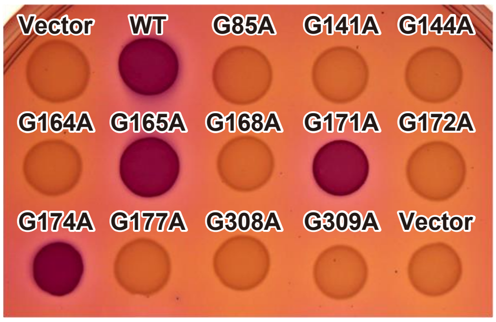

	TM	Nitrate uptake
Vector	▯	▯
WT	▯	++
G85A	TM2	▯
G141A	TM4	▯
G144A	TM4	▯
G164A	TM5	▯
G165A	TM5	++
G168A	TM5	▯
G171A	TM5	++
G172A	TM5	▯
G174A	TM5	++
G177A	TM5	▯
G308A	TM8	▯
G309A	TM8	▯

Supplementary Figure 5 Genetic analysis of the Gly mutants in TM2, TM4, TM5, and TM8.

Genetic analysis of the nitrate transport activities of NarK mutants of the conserved Gly residues in TM2, TM4, TM5, and TM8 by the "no-induction system". The results are summarized in the right panel.

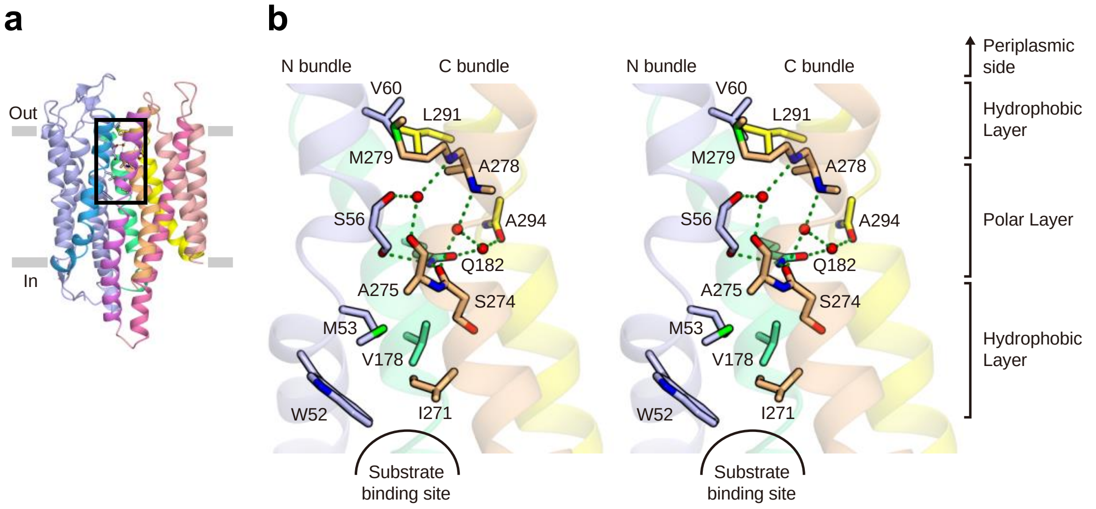

Supplementary Figure 6   Periplasmic interactions of NarK.

(**a**) Overview of the structure of NarK in the nitrate-bound occluded state (Mol A). The colors of the helices in the inward-open state correspond to those in Fig. 2, (a) and (b). A close-up view of the area indicated by the rectangle is provided in panel (b). (**b**) Stereo view of the interactions between the N and C bundles that occlude the substrate-binding site from the periplasmic side. TM1, TM5, TM7, and TM8 are shown by ribbon models in pale colors. The residues composing the two hydrophobic layers and the polar layers are shown in stick models. Water molecules are depicted by red spheres. Hydrogen bonds are represented by green dashed lines.

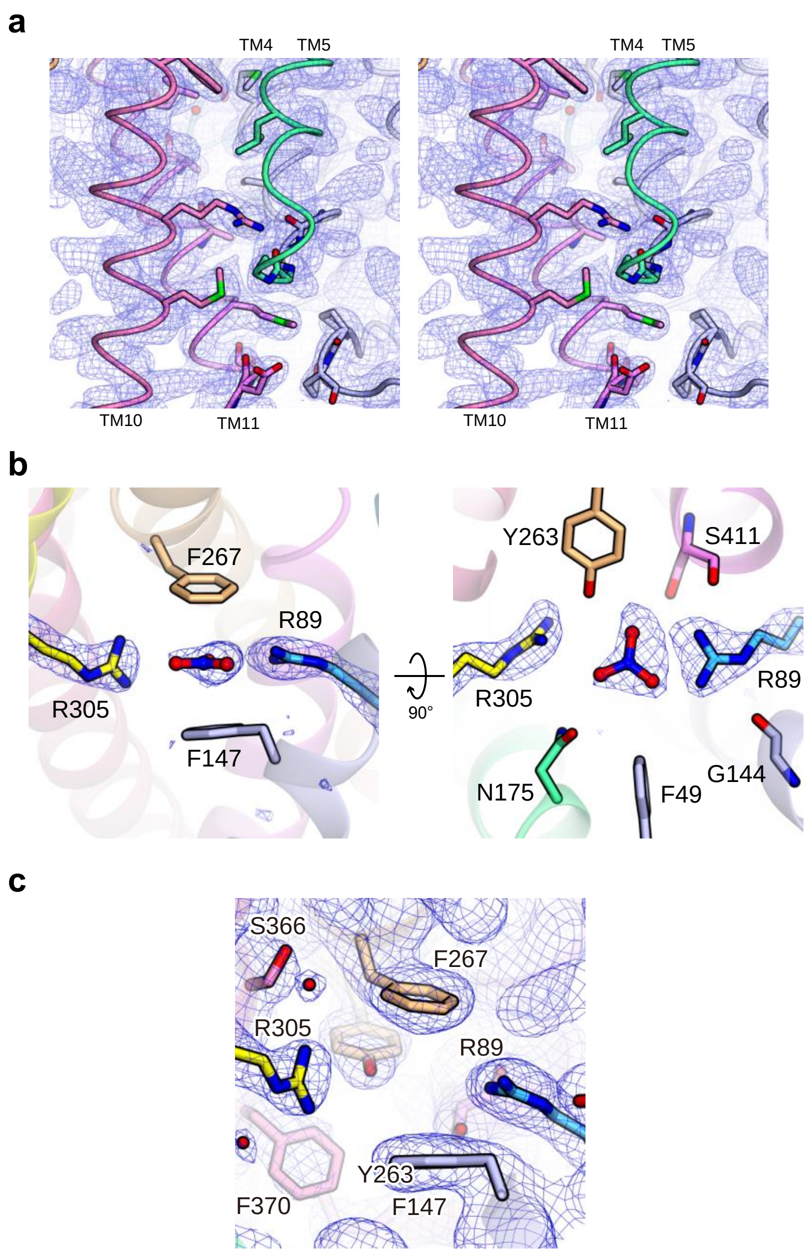

Supplementary Figure 7 Electron density maps of NarK.

(**a**) The $2mF_o$–DF_c electron map contoured at 1.1 σ around the cytosolic transport path of NarK, in the nitrate-bound occluded state (stereo view). (**b**) The mF_o–DF_c omit maps contoured at 4 σ around the substrate-binding site of the nitrate-bound inward-open state. The nitrate ion and the side chains of R89 and R305 were omitted in the map calculation. (**c**) The $2mF_o$–DF_c electron map contoured at 1.1 σ around the substrate-binding site of NarK, in the *apo* inward-open state.

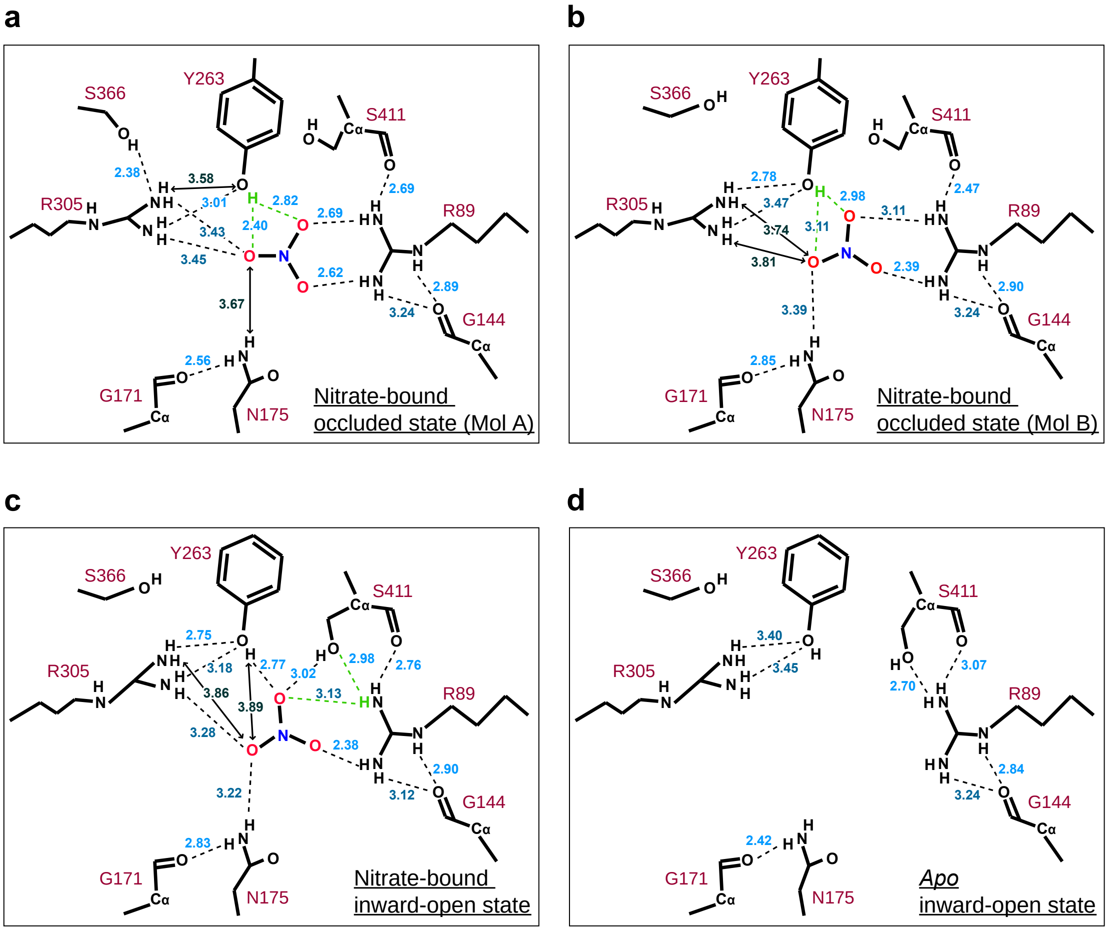

Supplementary Figure 8 Schematic drawings of the hydrogen bond geometry in the substrate-binding site of each NarK molecule.

(**a**), (**b**), (**c**), and (**d**) Alternative hydrogen-bonding configurations possible for (a) the nitrate-bound occluded state (Mol A), (b) the nitrate-bound occluded state (Mol B), (c) the nitrate-bound inward-open state, and (d) the *apo* inward-open state are shown. The potential hydrogen bonds and electrostatic interactions within 3.5 Å are depicted as black dashed lines. Distances over 3.5 Å are represented by black arrows. The bifurcated hydrogen bonds and the hydrogen atoms participating in them are colored light green.

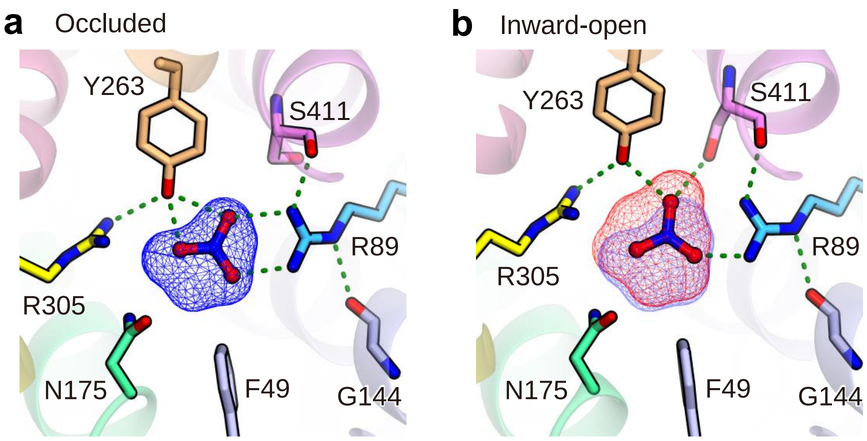

Supplementary Figure 9 Volumes of the cavity in the substrate-binding site.

(**a**) The cavity in the substrate-binding site of the occluded state. The blue mesh represents the surface of the cavity calculated by the program MSMS, using a 1.2 Å radius sphere as a probe. The green dashed lines represent the hydrogen bonds up to 3.1 Å. (**b**) The cavity in the substrate-binding site of the inward-open state. The red mesh represents the surface of the cavity, calculated as in panel (a). The cavity in the occluded state is also shown in a blue transparent mesh, for comparison.

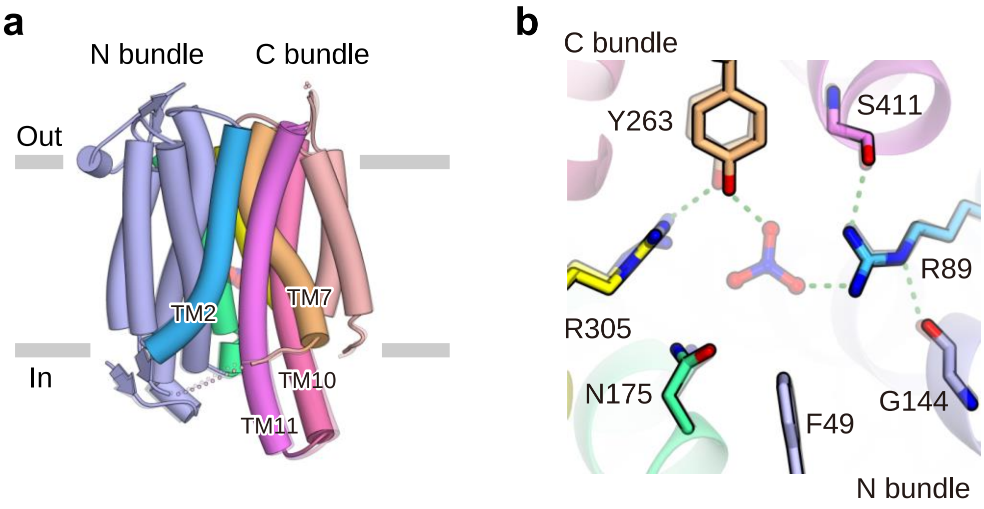

Supplementary Figure 10 Structure of NarK in the *apo* inward-open state.

(**a**) Overall structure of the *apo* inward-open state, shown as a cartoon model. (**b**) Close-up view around the substrate-binding site. The structure of the nitrate-bound inward-open structure, superimposed over all of the Cα atoms, is shown for comparison in both panels. The colors of the helices and residues correspond to those in Fig. 2. (a) and (b). The semi-transparent green dashed lines represent the hydrogen bonds found in the nitrate-bound inward-open state.

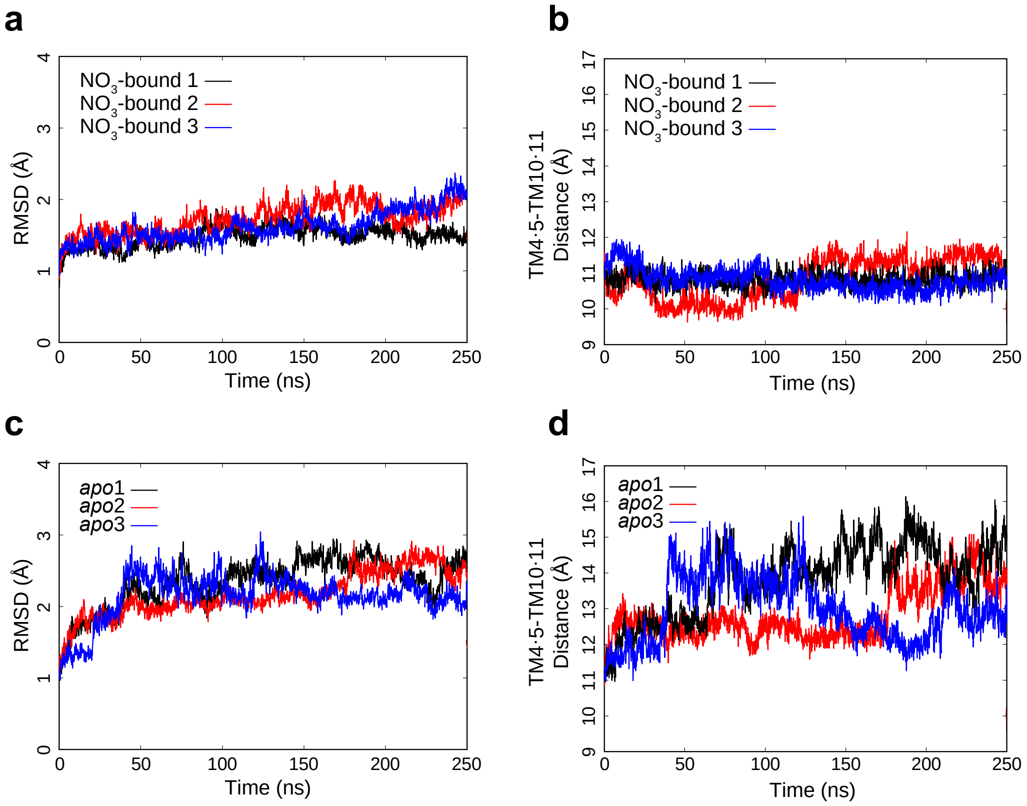

Supplementary Figure 11 MD simulations from apo and nitrate-bound occluded structures repeated three times.

The results of each of the three MD simulations are colored black, red, and blue. (**a**) and (**b**) MD simulations starting from the nitrate-bound occluded structure. (a) Plots of the RMS deviations of the overall Cα atoms from the initial crystal structure (nitrate-bound occluded state) as a function of time. (b) Plots of the TM4·5-TM10·11 distance, as in Fig. 6c, as a function of time. (**c**) and (**d**) MD simulations starting from the apo occluded structure. (c) Plots of the RMS deviations of the overall Cα atoms from the initial crystal structure (apo occluded state), as a function of time. (d) Plots of the TM4·5-TM10·11 distance, as in Fig. 6c, as a function of time. In each panel, the results colored in black are those shown in Fig. 6a and c.

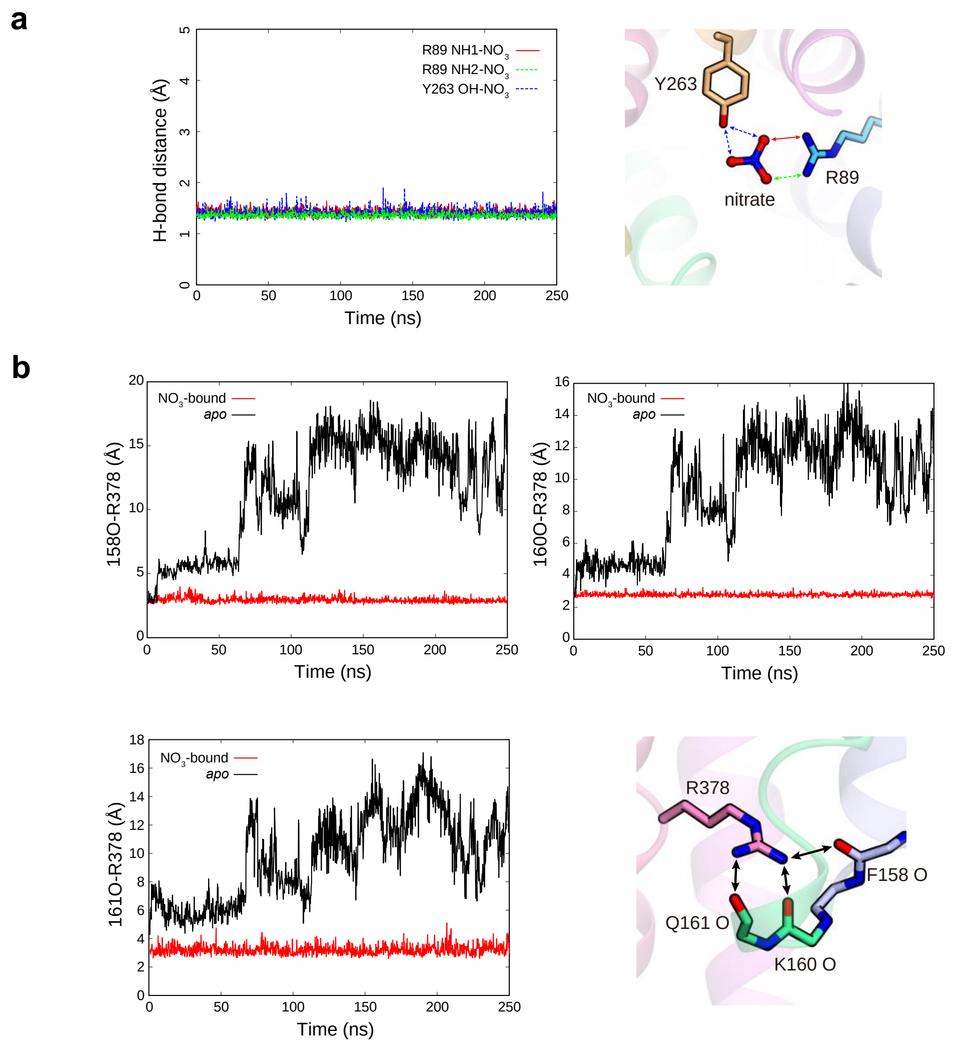

Supplementary Figure 12 The interaction distances in the substrate-binding site and the cytosolic pathway in the molecular dynamics simulations.

(**a**) Plots of the hydrogen-bond distances between the oxygen atoms of nitrate and the substrate-binding site, in the simulation starting from nitrate-bound occluded structures. The distances plotted in the graph are indicated by the arrows in the close-up view of the substrate-binding site, shown in the right panel. (**b**) Plots of the distances between the Arg378 side chain in TM10 in the C bundle and the loop connecting TM4 and TM5 in the N bundle, in the simulations starting from nitrate-bound occluded (red line) and apo occluded (black line) structures. In the bottom-right panel, the side chain of R378 and the main chains of F158, K160, and Q161 are depicted by stick models. The distances plotted in these graphs are indicated by the black arrows.

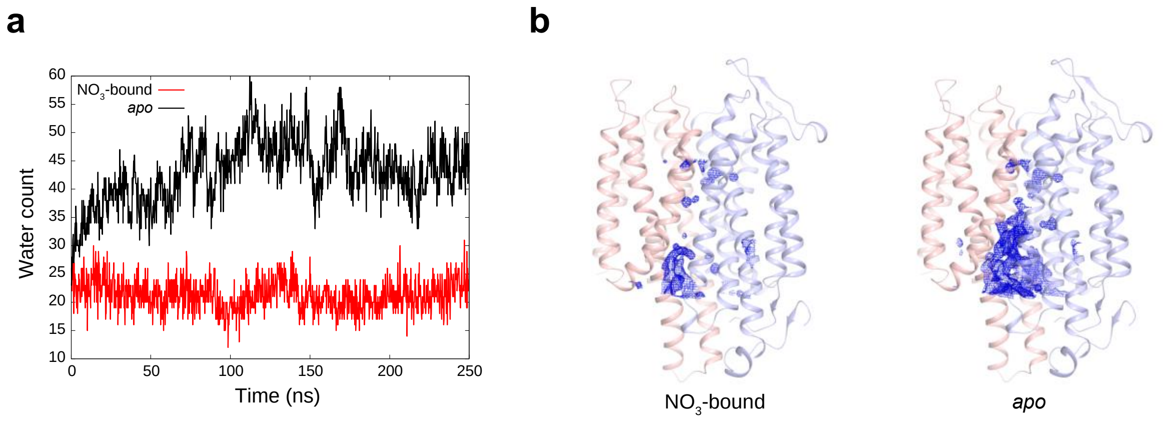

Supplementary Figure 13 Water molecules entering the cytoplasmic transport pathway in the *apo* occluded simulation.

(**a**) Plots of the number of water molecules entering the cytoplasmic transport pathway and the substrate-binding site, as a function of time. (**b**) The water probability density map around the substrate-binding site in the nitrate-bound (left panel) and *apo* (right panel) simulations, contoured at 0.001 molecules $Å^{-3}$ ns^{-1}. The probability was calculated by averaging over the 250 ns simulation.

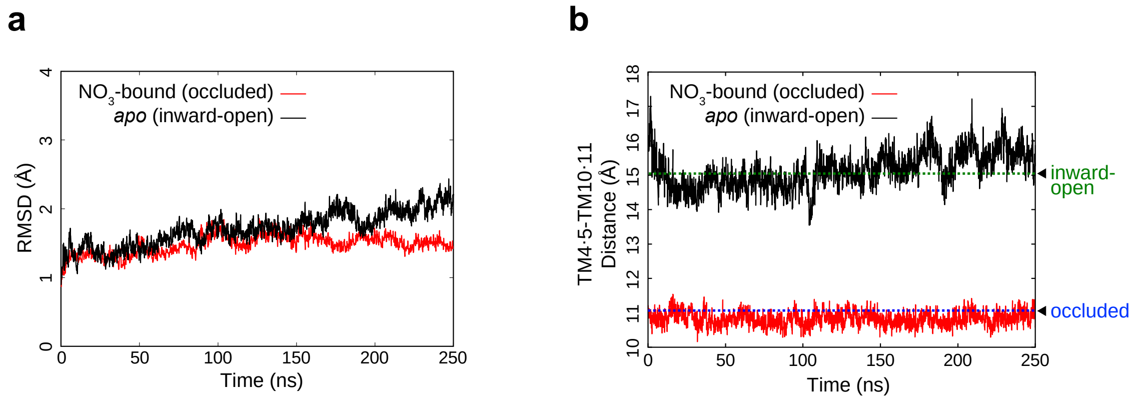

Supplementary Figure 14 MD simulation starting from the *apo* inward-open structure.

(**a**) Plots of the RMS deviations of the overall Cα atoms from the initial crystal structures (nitrate-bound occluded and *apo* inward-open states), as a function of time, during the simulations. (**b**) Plots of the TM4·5-TM10·11 distance, as in Fig. 6c, as a function of time, during the nitrate-bound occluded and *apo* inward-open simulations. The TM4·5-TM10·11 distances of two crystal structures (occluded and inward-open state) are shown by blue and green dashed lines, respectively, and indicated by black rectangles.

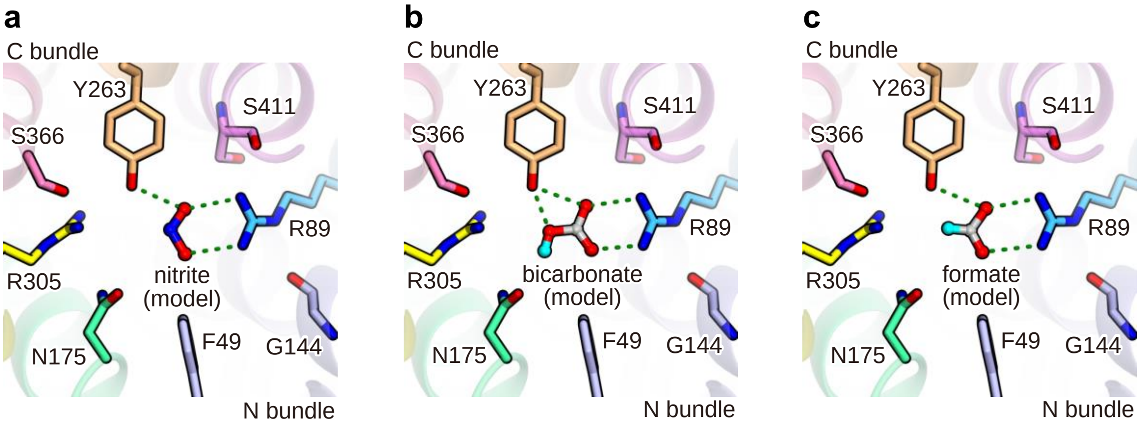

Supplementary Figure 15 Recognition models of nitrite and other anions by NarK.

(**a**), (**b**) and (**c**) The recognition models of (a) nitrite, (b) bicarbonate, and (c) formate by NarK, based on the present high-resolution nitrite-bound structures. The possible hydrogen bonds between nitrite and the R89 and Y263 side-chains are indicated by green dashed lines.

Supplementary Table 1. Primers for cloning *narK* gene

Name	Fw/Rv	Sequence
NarK (WT)	Fw	5'- GCGCCCATGGCCAGTCACTCATCCGCCCCCGAAAG -3'
	Rv	5'- GCGCCTCGAGTTTTTTAGAATGCCGACCATATAC -3'
NarK (G268A)	Fw	5'- GTATCTGGCAACCTTCGCCTCCTTCATCGGCTTC -3'
	Rv	5'- GAAGCCGATGAAGGAGGCGAAGGTTGCCAGATAC -3'
NarK (ΔG3)	Fw	5'- CTAGTGCTTCCACTTTCCAGATGATTTCCGTG -3'
	Rv	5'- CTTCCTGGCGCTGTTCCTGACCGCTGCGCTGG -3'
NarK (G408A)	Fw	5'- GACACGGCGGCGGCGCTGCCTTTCATCTCTG -3'
	Rv	5'- CAGAGATGAAAGGCAGCGCCGCCGCCGTGTC -3'
NarK (G418A)	Fw	5'- GATTGGCGCGATTGGTGCCTTCTTTATCCCGAAAG -3'
	Rv	5'- CTTTCGGGATAAAGAAGGCACCAATCGCGCCAATC -3'
NarK (R89K)	Fw	5'- GGTTTCTGGCGCGTTATTAAAGGTTCCATACTCCTTTATGC -3'
	Rv	5'- GCATAAAGGAGTATGGAACCTTTAATAACGCGCCAGAAACC -3'
NarK (R305K)	Fw	5'- GTTTATTGGTGCGCTGGCGGCTTCTGCAGGTGGTG -3'
	Rv	5'- CACCACCTGCAGAAGCCGCCAGCGCACCAATAAAC -3'
NarK (Y263F)	Fw	5'- GTGGATTATGAGCCTGCTGTTTCTGGCAACCTTCGG -3'
	Rv	5'- CCGAAGGTTGCCAGAAACAGCAGGCTCATAATCCAC -3'
NarK (F147A)	Fw	5'- CTTTGCTGGCGCGAACGCCGCATCCAGTATGG -3'
	Rv	5'- CCATACTGGATGCGGCGTTCGCGCCAGCAAAG -3'
NarK (F267A)	Fw	5'- GCTGTATCTGGCAACCGCCGGCTCCTTCATCGG -3'
	Rv	5'- CCGATGAAGGAGCCGGCGGTTGCCAGATACAGC -3'
NarK (G85A)	Fw	5'- CTGCCTTCGGTTTCTGCCGCGTTATTACGTG -3'
	Rv	5'- CACGTAATAACGCGGCAGAAACCGAAGGCAG -3'
NarK (G141A)	Fw	5'- CATTTCTCTGCTGTGCGCCTTTGCTGGCGCGAAC -3'
	Rv	5'- GTTCGCGCCAGCAAAGGCGCACAGCAGAGAAATG -3'
NarK (G144A)	Fw	5'- CTGTGCGGCTTTGCTGCCGCGAACTTCGCATCCAG -3'
	Rv	5'- CTGGATGCGAAGTTCGCGGCAGCAAAGCCGCACAG -3'
NarK (G164A)	Fw	5'- CCGAAACAGAAGCAGGCTGGCGCGCTGGGTCTG -3'
	Rv	5'- CAGACCCAGCGCGCCAGCCTGCTTCTGTTTCGG -3'
NarK (G165A)	Fw	5'- GAAACAGAAGCAGGGTGCCGCGCTGGGTCTGAATG -3'
	Rv	5'- CATTCAGACCCAGCGCGGCACCCTGCTTCTGTTTC -3'
NarK (G168A)	Fw	5'- GCAGGGTGGCGCGCTGGCTCTGAATGGTGGTCTG -3'
	Rv	5'- CAGACCACCATTCAGAGCCAGCGCGCCACCCTGC -3'

NarK (G171A)	Fw	5'- CGCGCTGGGTCTGAATGCTGGTCTGGGCAACATGG -3'
	Rv	5'- CCATGTTGCCCAGACCAGCATTCAGACCCAGCGCG -3'
NarK (G172A)	Fw	5'- CGCTGGGTCTGAATGGTGCTCTGGGCAACATGGGC -3'
	Rv	5'- GCCCATGTTGCCCAGAGCACCATTCAGACCCAGCG -3'
NarK (G174A)	Fw	5'- CTGAATGGTGGTCTGGCCAACATGGGCGTCAGC -3'
	Rv	5'- GCTGACGCCCATGTTGGCCAGACCACCATTCAG -3'
NarK (G177A)	Fw	5'- GGTCTGGGCAACATGGCCGTCAGCGTCATGCAG -3'
	Rv	5'- CTGCATGACGCTGACGGCCATGTTGCCCAGACC -3'
NarK (G308A)	Fw	5'- CTGGCGCGTTCTGCAGCTGGTGCATTATCTGAC -3'
	Rv	5'- GTCAGATAATGCACCAGCTGCAGAACGCGCCAG -3'
NarK (G309A)	Fw	5'- GGCGCGTTCTGCAGGTGCTGCATTATCTGACCG -3'
	Rv	5'- CGGTCAGATAATGCAGCACCTGCAGAACGCGCC -3'

NcoI and XhoI restriction sites are colored blue and red, respectively.

Supplementary Table 2. Primers for gene knock-outs.

Name	Knock-out gene	Sequence
NAP1	Δ*napA-napB*	5'- TATGAAAGCTAACGCCGTTGCGGCCGCTGCGGCGGCTGCCGTGTAGG CTGGAGCTGCTTC -3'
NAP2	Δ*napA-napB*	5'- GGTAAAGGTATTCCCCACGATTGGCGCGGTATCGGCCTGCCATATGA ATATCCTCCTTA -3'
NAR1	Δ*narZ-narU*	5'- AATAGTCGTTATCTTTTGCGCGACTGGAAACCAGAAAATCGTGTAGG CTGGAGCTGCTTC -3'
NAR2	Δ*narZ-narU*	5'- GATCGCGACCTTCATCATCCAGCCAGTTAACGTTCTTCATCATATGAA TATCCTCCTTA -3'
